# Current Advances in *Coptidis Rhizoma* for Gastrointestinal and Other Cancers

**DOI:** 10.3389/fphar.2021.775084

**Published:** 2022-01-03

**Authors:** Luying He, Zhangfeng Zhong, Man Chen, Qilian Liang, Yitao Wang, Wen Tan

**Affiliations:** ^1^ School of Pharmacy, Lanzhou University, Lanzhou, China; ^2^ Macau Centre for Research and Development in Chinese Medicine, Institute of Chinese Medical Sciences, University of Macau, Macao SAR, China; ^3^ Oncology Center, Affiliated Hospital of Guangdong Medical University, Zhanjiang, China

**Keywords:** coptidis rhizoma, medicinal plant, gastrointestinal cancer, omics, clinical research

## Abstract

*Cancer* is a serious disease with an increasing number of reported cases and high mortality worldwide. Gastrointestinal cancer defines a group of cancers in the digestive system, e.g., liver cancer, colorectal cancer, and gastric cancer. *Coptidis Rhizoma* (*C. Rhizoma*; Huanglian, in Chinese) is a classical Chinese medicinal botanical drug for the treatment of gastrointestinal disorders and has been shown to have a wide variety of pharmacological activity, including antifungal, antivirus, anticancer, antidiabetic, hypoglycemic, and cardioprotective effects. Recent studies on *C. Rhizoma* present significant progress on its anticancer effects and the corresponding mechanisms as well as its clinical applications. Herein, keywords related to *C. Rhizoma*, cancer, gastrointestinal cancer, and omics were searched in PubMed and the Web of Science databases, and more than three hundred recent publications were reviewed and discussed. *C. Rhizoma* extract along with its main components, berberine, palmatine, coptisine, magnoflorine, jatrorrhizine, epiberberine, oxyepiberberine, oxyberberine, dihydroberberine, columbamine, limonin, and derivatives, are reviewed. We describe novel and classic anticancer mechanisms from various perspectives of pharmacology, pharmaceutical chemistry, and pharmaceutics. Researchers have transformed the chemical structures and drug delivery systems of these components to obtain better efficacy and bioavailability of *C. Rhizoma*. Furthermore, *C. Rhizoma* in combination with other drugs and their clinical application are also summarized. Taken together, *C. Rhizoma* has broad prospects as a potential adjuvant candidate against cancers, making it reasonable to conduct additional preclinical studies and clinical trials in gastrointestinal cancer in the future.

## Introduction

*Coptidis Rhizoma* (*C. Rhizoma*; Huanglian, in Chinese) is a common botanical drug which has a long history in Asia, especially in China. It is constituted by the dried root of three Coptis species, namely, *Coptis chinensis* Franch. (Weilian in Chinese), *Coptis deltoidea* C.Y. Cheng et Hsiao (Yalian), and *Coptis teeta* Wall. (Yunlian), in the *Chinese Pharmacopoeia Edition 2020*. Other native species of Coptis, such as *Coptis trifolia* Salisb and *Coptis japonica* Makino, are distributed in other regions of the world (Wang et al., 2014a). As recorded in *Shennong’s Materia Medica*, *C. Rhizoma* is used to treat high fever, vomiting, diarrhea, abdominal fullness, jaundice, toothache, and eczema, in the traditional dosage form, i.e., powder, pill, decoction, or tablet (Wang et al., 2019). In the past decade, various studies have been conducted to unravel its pharmacological activities and the possible underlying mechanisms of its action. Its wide pharmacological activities include antiviral, antibacterial, antifungal, antihepatic steatosis, antiatherosclerotic, antiarrhythmic, antihypertensive, cardioprotective, antidiabetic, anti-inflammatory, antioxidative, neuroprotective, and anticancer effects (Meng et al., 2018; Lyu et al., 2021).

*Cancer* is a leading cause of death around the world. Urgent strategies are required to overcome this disease given the population growth and ageing (Omran, 1971; Bray et al., 2018). Gastrointestinal cancer defines a group of cancers that affect the digestive system, including gastric cancer, colorectal cancer (CRC), liver cancer, esophageal cancer, pancreatic cancer, anal cancer, bile duct cancer, gastrointestinal stromal tumor, gallbladder cancer, and small intestine cancer. In particular, digestive system cancers are the first among estimated cancer-related deaths (27.82%, 169,280/608,570) and the second among estimated new cases (17.81%, 338,090/1,898,160) diagnosed at all cancer sites according to the *Cancer* Statistics 2021, United States (Siegel et al., 2021). As a main treatment for cancer, chemotherapy often indistinguishably kills healthy cells and exerts toxic effects on patients (Zaimy et al., 2017). In addition, cancer metastasis and the development of multidrug resistance (MDR) might occur. Despite the enormous amount of research performed to identify the complex causes of these obstacles and various treatment options, there is still a considerable amount research needed on natural products (Hausman, 2019), especially the conventional botanical drug *C. Rhizoma* for the treatment of gastrointestinal disorders. The antitumor effects of *C. Rhizoma* have been known for many years. The corresponding mechanisms involve the exacts as well as the main compounds, such as berberine, palmatine, coptisine, magnoflorine, jatrorrhizine, epiberberine, oxyepiberberine, oxyberberine, columbamine, and limonin. Moreover, the benefits of *C. Rhizoma* in antiviral and anti-inflammatory effects is closely associated with its anticancer effects, when administered as its traditional prescription (Tang et al., 2009). After all, inflammatory cells and functional polymorphisms of genes encoding inflammatory cytokines are associated with tumor growth and progression and determine whether or not an effective host antitumor response could be achieved in the susceptibility and severity state of cancer (Balkwill and Mantovani, 2001). Given the great potential of *C. Rhizoma* in cancer treatment, researchers have systematically discussed its druggability in 2015 (Wang et al., 2015). Recent studies have focused on the application of *C. Rhizoma* in gastrointestinal cancer.

## Chemical Constituents of *Coptidis Rhizoma*

**FIGURE 1:**
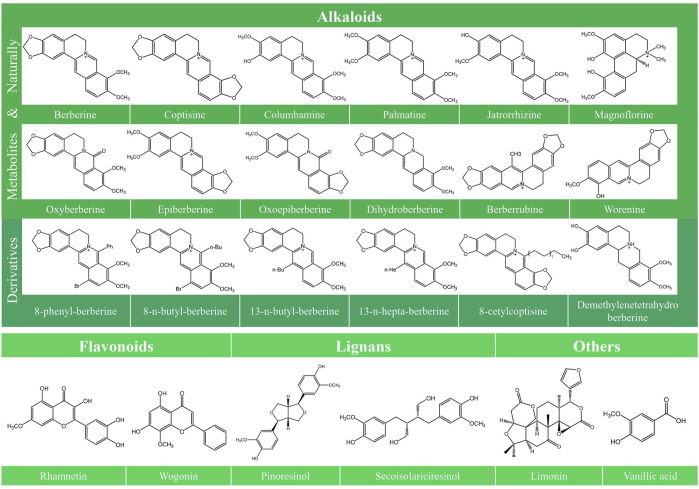
Chemical structures of the constitutes derived from *Coptidis Rhizoma*. The chemical structures of the constitutes derived from *Coptidis Rhizoma* are shown, including Berberine, Coptisine, Columbamine, Palmatine, Jatrorrhizine, Magnoflorine, Oxyberberine, Epiberberine, Oxoepiberberine, Dihydroberberine, Berberrubine, Worenine, 8-phenyl-berberine, 8-n-butyl-berberine, 13-n-butyl-berberine, 13-n-hepta-berberine, 8-cetylcoptisine, Demethylenetetrahydroberberine, Rhamnetin, Wogonin, Pinoresinol, Secoisolariciresinol, Limonin, and Vanillic acid.

Various typical constituents and abundant secondary metabolites were found in *C. Rhizoma*, including alkaloids, flavonoids, lignans, phenolic acids, phenylpropanoids, saccharides, and steroids (Meng et al., 2018). Recent studies have improved our understanding of the antitumor activities of *C. Rhizoma*. Alkaloids are the main components of *C. Rhizoma*, and the anticancer studies involving *C. Rhizoma* have mostly concerned on the berberine-type and oxyberberine-type components (Meng et al., 2018). According to their chemical structures, alkaloids can be divided into different subtypes: berberine-type alkaloids, oxyberberine-type alkaloids, methyl-berberine type alkaloid, benzylisoquinolines, benzophenanthridines, protoberberine-type alkaloids, phenethylamines, aporphine, isoquinolines, and other nitrogen-containing molecules (Chen et al., 2008; Li et al., 2012; Yang et al., 2014). Other constituents include lignans with numerous subclasses (Meng et al., 2018), abundant flavonoids (Chen et al., 2012), and phenylpropanoids with various molecular weight and structures. However, there are few published studies investigating the anticancer effects of the latter three compounds isolated from *C. Rhizoma*. Limonin, another compound isolated from *C. Rhizoma*, has been indicated to exert broad anticancer effects in a variety of human cancer cells (Fan S. et al., 2019). The chemical structure of the compounds together with their anticancer activities described in this review is shown in Figure 1.

## Antitumor Effects and Corresponding Mechanisms

### *C. Rhizoma* Extracts and Its Main Component Berberine

There are many studies describing the antitumor effects and the corresponding mechanisms of *C. Rhizoma* extract and those of berberine. Common mechanisms include induction of apoptosis, regulation of signal transduction, arresting of the tumor cell cycle, inhibition of tumor migration and invasion, and epigenetic regulation. In recent years, novel mechanisms have emerged, such as regulating autophagy, immunity, inflammation, gut microbiota, and microRNA (miRNA). The current research hotspot is the relationship of tumor, immunity, and inflammation.

#### The Novel Mechanisms

Novel mechanisms of action of berberine, including autophagy, immunity, inflammation, modification of the gut microbiota and miRNA, have been the areas of research in recent studies of *C. Rhizoma*. These mechanisms are briefly elaborated below.

##### Autophagy

Autophagy is a multistep process, involving the cytoplasmatic autophagosome (Lee et al., 2019). The anticancer mechanisms related to autophagy of berberine are found in a variety of cancer types. mTOR is a major regulator of cell metabolism and are closely associated with cancers. It consists of mTORC1 and mTORC2 (Kim and Guan, 2015). Increased mTORC1 activity contributes to the survival of cancer cells (Martínez-Carreres et al., 2019) and overactivation of protein kinase B (AKT)/mTORC1 signaling leads to excessive proliferation of cancer cells and impairs autophagy-mediated cell death (Erazo et al., 2016). Berberine activates cytostatic autophagy by upregulating the expression of autophagy-related proteins, such as LC3-II, *p*-ULK1, especially Beclin-1 and suppressing the phosphorylation of AKT, extracellular signal-regulated kinase (ERK), c-Jun N-terminal kinase (JNK), p38, mTOR and p70S6K in gastric cancer (BGC-823) cells. *In vivo* model, Beclin-1 and LC3-II were increased in tumor tissue, the same inhibitions of phosphorylation were as the above (Zhang Q. et al., 2020). Acute lymphoblastic leukemia (ALL) is a common type of leukemia (Khwaja et al., 2016; Papaemmanuil et al., 2016). In ALL, berberine promotes autophagic cell death and significantly ameliorates the conditions of ALL by increasing LC3-II and Beclin-1 and inactivating the AKT/mTORC1 signaling pathway (Liu et al., 2020a). Murine double minute 2 (MDM2), a proto-oncogene, has been found to be upregulated in a variety of cancers and interacts with p53 (Oliner et al., 1992; Bueso-Ramos et al., 1993), thereby promoting degradation of p53 and inhibiting p53-mediated transactivation (Momand et al., 1992; Haupt et al., 1997). At the transcriptional and post-transcriptional levels, berberine was found to induce autophagy by downregulating MDM2 expression in p53-deficient leukemic cells and contributed to the apoptosis-promoting effect in p53-deficient leukemic cells (Liu et al., 2020c). Temozolomide is often used to treat glioblastoma (Delgado-López and Corrales-García, 2016; Nørøxe et al., 2016; Paolillo et al., 2018), and berberine reduces drug resistance of temozolomide by augmenting autophagy via downregulating the activation of the ERK1/2 signaling pathway. *In vivo* model, berberine increases glioblastoma sensitivity to temozolomide through the same signaling pathways (Qu et al., 2020). The emerging photodynamic therapy (PDT), which is based on photosensitizer-mediated oxidative cytotoxicity, is regarded as a promising approach in the treatment of cancers. Berberine-associated PDT exerts antiproliferative effects on human malignant melanoma cells, by inducing cleaved Caspase-3-mediated apoptosis, increasing LC3-related autophagy, activating endoplasmic reticulum stress, and promoting a dramatic increase in Reactive Oxygen Species (ROS) (Fang et al., 2021). However, autophagy also plays a negative role in cancer cells. *Cancer* cells can avoid apoptosis through regulation of autophagy, consequently causing drug resistance and enhancing tumor cell viability (Sui et al., 2014). Hepatocellular carcinoma (HCC) caused by the hepatitis C virus is a deadly disease that induces autophagy and is highly refractory to chemotherapy. Berberine can augment cell apoptosis and necrosis by inhibiting autophagy via targeting ROS and LC3-II in HCC (Huh-7) cells infected with hepatitis C virus RNA (Tai et al., 2020). In addition, berberine inhibits autophagy by downregulating the expression of phosphatase and tensin homolog (PTEN) to increase the phosphorylation of Akt and mTOR. In the case of breast cancer, berberine acts as an autophagy inhibitor to inhibit autophagosome formation in doxorubicin (DOX)-resistant breast cancer (MCF-7) cells and blocks accumulation of LC3-II, and increases expression of the p62 protein, finally reducing cell proliferation and reversing DOX resistance both *in vitro* and *in vivo* (Wang et al., 2020). Autophagy is an attractive mechanism that has been identified in recent years. Many unknown areas and details, including a description of the definitive effects and mechanisms of *C. Rhizoma* on cancer treatment, still need to be resolved.

##### Immunity

Immune escape is a major feature of a variety of cancers (Dunn et al., 2004). Berberine and some components of *C. Rhizoma* have immunomodulatory effects (Li H. et al., 2014). The levels of granulocytic myeloid-derived suppressor cell (G-MDSC), a subset of suppressive myeloid cells, consistently increase in tumor-bearing mice and are closely associated with tumor-induced granulocytic hyperplasia, a process related to tumor vasculogenesis and immune escape (Talmadge and Gabrilovich, 2013). Berberine significantly suppresses acute/chronic hepatic damage in mice by regulating the G-MDSC-like population mediated in part, by the interleukin (IL)-6/STAT3 signaling pathway. This is also involved in the regulation of the gut microbial community represented by the increased *Akkermanisa muciniphila* (Li S. et al., 2020). Berberine exerts antitumor activity in diffuse large B-cell lymphoma, a subtype of non-Hodgkin lymphoma by modulating the c-myc/CD47 axis. CD47 is a target for improving treatment efficacy, and the overexpression of CD47 is related to immune escape (Casey et al., 2016; Takimoto et al., 2019; Eladl et al., 2020). Berberine, a suppressor of CD47, downregulates CD47 mRNA by suppressing c-Myc expression (Ren et al., 2021). The suppression of CD47 enhances the phagocytosis of macrophages, which contributes to eliminate diffuse large B-cell lymphoma cells both *in vitro* and *in vivo*. In addition, berberine can improve the efficiency of the anti-CD47 antibody and rituximab on cell phagocytosis (Chao et al., 2010; Weiskopf et al., 2016; Schürch et al., 2019). Berberine is beneficial in immunochemotherapy based on rituximab in combination with anti-CD47 in diffuse large B-cell lymphoma (Harris et al., 2020; Ren et al., 2021).

Blocking the programmed cell death-1 (PD-1)/programmed cell death ligand-1 (PD-L1) has become the main strategy in cancer immunotherapy (Iwai et al., 2017). In non-small cell lung cancer (NSCLC), berberine reduces the expression of PD-L1 and promotes antitumor immunity by inhibiting the activity of COP9 signalosome 5, along with activation of tumor-infiltrating T cells (Liu Y. et al., 2020). Furthermore, berberine shows a synergistic effect against breast cancer both *in vitro* and *in vivo*. This combination activates the immune system, regulates levels of intestinal microbial metabolites, activates the mitochondrial apoptotic signaling pathway, and the Fas death receptor pathway (Ma et al., 2020). Berberine also functions as a potent antioxidant and immunomodulatory agent, having a protective function on the liver. Berberine reverses the elevation of serum and tissue stress induced by DOX, as well as in tissue inflammatory mediators and serum cytokine levels. Some cytochrome P450s (CYP), including 2B1, 2B2 and 2E1, also show significantly reduced expression upon berberine treatment (Sun B. et al., 2020). Additionally, the immune modulation of berberine on gastrointestinal cancer is evident in inflammatory bowel disease (IBD) and in the associated CRC, which is described in the following paragraph.

##### Inflammation

Chronic inflammation is known to promote carcinogenesis. It was estimated approximately 15–20% of all cancer-related deaths are related to inflammatory processes and the underlying infections (Eiró and Vizoso, 2012). *C. Rhizoma* and its active component berberine suppress inflammatory processes to ameliorate development of IBD associated with CRC. The anti-inflammatory effects of berberine include the regulation of cell cycle, induction of apoptosis, and prevention of leukocyte migration by inhibiting classic inflammatory pathways and the expression of various chemokines (Xu et al., 2020). To attenuate inflammation in the early phase, berberine interacts with TLR4 and interferes with the TLR4/MyD88/NFκB signaling pathway (Gong et al., 2019a). Researchers identified NIMA-related kinase 7 (NEK7) as a new target of inflammatory diseases related to the nucleotide binding domain (NOD)-like receptor protein 3 (NLRP3) (He et al., 2016; Xu et al., 2016). Berberine acts on NEK7 protein through the hydrogen bond to affect the NEK7−NLRP3 interaction and prevents IL-1β release to exert anti-inflammatory effect both *in vitro* and *in vivo* (Zeng et al., 2021). At the same time, berberine is able to directly inhibit the functions of pro-inflammatory Th1 and Th17 cells and their differentiation, which together indirectly reducing Th cell-mediated inflammation by regulating other cells, such as Tregs, that contribute to autoreactive inflammation (Ehteshamfar et al., 2020).

IBD is a recurrent gastrointestinal inflammatory disease that includes two different conditions: ulcerative colitis (UC) and Crohn’s disease (CD) (Gunnarsson et al., 2012). They are associated with a higher risk of colitis-associated CRC with poor prognoses (Watanabe et al., 2011; Zhen et al., 2018). CRC ranks the top three in cancers around the world (Mármol et al., 2017). The highly conserved RNase III enzyme Dicer, an essential component of the RNA interference pathway (Foulkes et al., 2014), is decreased and promotes carcinogenesis (Kumar et al., 2007). The decrease in Dicer expression could increase the levels of cytosolic DNA and IL-6 mRNA under oxidative stress in inflammatory bowel tissues, while berberine alleviates colitis by recovering Dicer expression to exert a preventive effect on colitis-associated tumorigenesis (Wu et al., 2020). In the dextran sulfate sodium (DSS)-induced colitis model, berberine exerts its therapeutic effects by activating the mTORC1 signaling pathway to enhance the number of Treg cells and regulate the levels of intestinal microbiota-associated tryptophan metabolites and the activation of the aryl hydrocarbon receptor (Li Q. et al., 2019; Jing et al., 2021). Similarly, berberine downregulates the levels of COX-2 mRNA, protein, as well as the concentration of prostaglandin E2 in the CRC (HT-29) cells (Tai and Luo, 2003). It attenuates the expression of COX-2 and PGE2 to decrease JAK2 and STAT3 phosphorylation as well as matrix metalloproteinase (MMP)-2 and MMP-9 expression (Liu X. et al., 2015). Berberine also exerts antiproliferative activity by participating in inflammatory response-driven epidermal growth factor receptor (EGFR) signaling pathway, thereby preventing the progression of colitis-associated CRC (Li D. et al., 2017). Cytosolic phospholipase A2a (PLA2G4A), an enzyme that hydrolyzes phosphatidylcholine to lysophosphatidylcholine, decreases in a colitis mouse model and in the inflammatory response of RAW 264.7 macrophages. Berberine binds to PLA2G4A directly and inhibits PLA2G4A activity by suppressing the MAPK/JNK signaling pathway to ameliorate colon inflammation (Zhai et al., 2020).

Berberine could also improve inflammatory status by downregulating cytokines IL-1*β* and tumor necrosis factor (TNF)-*α* and upregulating IL-4 in the serum and colon tissue of UC rats through related signaling pathways (Jiang et al., 2021). Berberine is capable of preventing the proinflammatory cytokines producing in colitis (Zhou and Mineshita, 2000; Kawashima et al., 2004; Lee et al., 2010; Zhang et al., 2011; Yan et al., 2012). The relevant mechanism might be the inhibition on proinflammatory cytokines production through the activation of MAPK and NF-κB in lipopolysaccharides (LPS) -induced macrophages (Lee et al., 2010). In addition, berberine exerts a protective effect on UC by regulating the interaction between enteric glial cells (EGCs) and intestinal epithelial cells-immune cells by exerting modulatory effects on the apoptosis of EGCs and the expression of substance *p* and glial-derived neurotrophic factor (Li et al., 2020b). Furthermore, berberine can also suppress mucosal inflammation by decreasing oncostatin M (OSM) to treat chronic UC. The effects include attenuating intestinal inflammation, modulating pathological changes and interaction between intestinal stromal cells and immune cells. It is closely associated with the inhibition of phosphorylated OSM-mediated signal pathway, such as JAK-STAT, AKT, and MAPK (Li et al., 2020c). Furthermore, inhibition of cytokines expression, like TNF-α、transforming growth factor-β (TGF-β), IL-1, IL-1β, IL-6, IL-12, and interferon (IFN)-γ, decrease the expression of IL-4 and IL-10 at mRNA levels and the inhibition of phosphorylation of STAT3 and NF-κB p65 are implicated in the anti-inflammatory activity of berberine (Zhu L. et al., 2019). Berberine combined with carboxylmethyl chitosan by arylboronic ester can significantly improve the symptoms of colitis and colon damage by regulating IL-6 expression and remodeling the intestinal microbiota (Zhao L. et al., 2021).

The occurrence of breast cancer is closely related to cytokines that alter the microenvironment of inflammatory tumors. Berberine can reduce the migratory ability of breast cancer (MDA-MB-231) cells in an *in vitro* scratch model, thereby prolonging the wound healing time. In addition, berberine inhibits the phosphorylation of c-Jun and c-Fos. LPS treatment can increase the expression of cytokines, and berberine effectively reduces the expression of IL-6 and TNF-α. Berberine also suppresses the activation of NF-κB by preventing IκBα from degradation (Zhao and Zhang, 2020). In addition to inflammation, the gut microbiota is another aspect of berberine regulation on gastrointestinal cancer, which has been addressed in extensive studies in recent years.

##### Gut Microbiota

The study of gut microbiota has suggested the inhibitory effects of berberine on CRC. A disruption of the balance of the intestinal flora is characterized by decreased community diversity and decreased abundance of beneficial bacteria, and is a cause of IBD. The mycobiota dysbiosis signature plays an important role in the pathogenesis of CRC (Gao et al., 2017). It is known that one of the mechanisms of berberine treatment of intestinal inflammation is associated with its antibacterial activity (Habtemariam, 2016). A study showed that berberine altered the metabolic and composition of the gut microbiota in azoxymethane/DSS treated mice (Ren et al.). *Actinobacteria* and *Verrucomicrobia* at the phylum level, and some pathogenic species*,* including *f_*Erysipelotrichaceae and *Alistipes* at the genus level, are obviously decreased. Meanwhile, some short-chain fatty acid producing bacteria, such as *Alloprevotella*, *Flavonifractor*, and *Oscillibacter*, are increased (Chen YX. et al., 2020). In the case of gut inflammation-associated disease, berberine can decrease harmful bacteria, such as *Escherichia coli* and enterococci bacteria, and increase Lactobacilli and Bifidobacteria (Habtemariam, 2020). For example, berberine elevates lactic acid-producing bacteria and carbohydrate hydrolysis bacteria, and reduces conditional pathogenic bacteria to treat colonic damage in DSS-induced UC mice (Liao et al., 2020). Furthermore, berberine alleviates DSS-induced colitis by activating AhR, which adjusts the tryptophan metabolite levels associated with the gut microbiota (Jing et al., 2021). Meanwhile, berberine mediates the number of Treg and Th17, two inflammation-related cells, by regulating the intestinal flora in the colon, which works as a therapy for UC (Cui et al., 2018). Another anticancer mechanism of berberine is to promote butyrate production in the gut microbiota. Oral berberine increases the abundance of butyrate-producing bacteria, indirectly changing the composition of intestinal bacterial in mice. Berberine indirectly increases butyrate by inhibiting NADH and bacterial ATP production, hence the levels of phosphotransbutyrylase/butyrate kinase and butyryl-CoA (acetate-CoA transferase) increase (Wang et al., 2017). Furthermore, berberine could alter fecal metabolites to modify the gut microbiota in intestinal mucositis induced by 5-fluorouracil. In addition, berberine significantly increases butyrate and glutamine levels in feces. Meanwhile, berberine decreases *Proteobacteria* and enriches the abundance of *Firmicutes* at the phylum level, along with increasing the proportion of *unclassified_f_*Lachnospiraceae*, unclassified_f_*Porphyromonadaceae*, Lactobacillus, and unclassified_o_Clostridiales* at the genus level (Chen et al., 2020a). Given that botanical drugs are characterized by multiple components with multiple effects from a holistic point of view, modulation of the gut microbiota foresees a much larger role for the effects of *C. Rhizoma* on carcinogenesis.

##### miRNA

miRNAs are small non-coding RNA molecules of around 20 nucleotides in length (Ambros, 2004; Bartel, 2004; Gu and Zhou, 2021). miRNAs are capable of suppressing target gene expression via binding to its target mRNA, causing mRNA cleavage or translation inhibition (Wightman et al., 1993; O’carroll and Schaefer, 2013). miRNAs act as essential regulatory elements in many aspects of biological processes and various human diseases, and most miRNAs are dysregulated in cancers (Filipowicz et al., 2008). Dysregulation of miRNAs is usually caused by defects in the miRNA biogenesis pathway (Gurtner et al., 2016). However, some miRNA are reported to directly participate in the formation of cancers and might act as tumor suppressors (e.g. let-7, miR-15a and miR-16–1) or as oncogenes (e.g. the miR-17∼92 cluster, miR-155, and miR-21) (Calin and Croce, 2006; Zhang et al., 2007; Pereira et al., 2013). Berberine was shown to modulate miRNA to affect cell cytotoxicity, apoptosis, and invasion in a variety of multiple cancers (Filipowicz et al., 2008). In multiple myeloma, berberine increases Set9, which damages and suppresses NF-κB, resulting in the decline of miR-21 and Bcl-2 levels, and stimulating ROS generation and apoptosis (Hu et al., 2013). Berberine decreases IL-6 and STAT3 to suppress miR-21 levels, resulting in the upregulation of programmed cell death 4 (PDCD4) (Luo et al., 2014; Jiang et al., 2015; Ayati et al., 2017). The miR-106b/25 cluster participates in numerous cancer-associated signaling pathways and tumorigenesis as an oncogene. Berberine downregulates miR-106b/25 in multiple myeloma cells (Gu et al., 2017).

In HCC, berberine upregulates miR-21-3p to modulate the expression of methionine adenosyltransferase 2A and methionine adenosyltransferase 2B, leading to cell apoptosis and viability and reduction of proliferation (Lo et al., 2013). miR-22-3p is decreased in HCC and berberine increases its expression to suppress cell proliferation by targeting Sp1 (Chen J. et al., 2016). Berberine also induces miR-23a expression and suppresses NEK6, which is a negative regulator of p53 in human cancers (Wang et al., 2014b). The combination of berberine and heat shock protein 90 (Hsp90) inhibitors has synergistic antiproliferative effects on cell growth arrest by suppressing the overexpression of CDK4 as that of miRNA-296-5p, which leads to the activation of the Pin1-β-catenin-cyclin D1 signaling pathway in CRC (Su et al., 2015). Berberine also inhibits miR-21 expression by mediating the miR-21-integrin β4-PDCD4 pathway in CRC (HCT 116) cells (Lü et al., 2018). As for gastric cancer, berberine suppresses cell invasion by upregulating miR-203 which targets Bcl-w, resulting in sensitizing cisplatin-resistant cells to initiate a caspase-dependent apoptosis (You et al., 2016). Some studies have also reported that miRNA target genes are associated with the regulation of the cell cycle, the Ras signaling pathway, and the JAK-STAT signaling pathway in gastric cancer (SGC-7901) cells (Yang et al., 2018). miR-212 is a cancer-related miRNA with dual functions, acting as a tumor suppressor or an oncogene. Overexpression of miR-212 is associated with poor outcomes in patients with esophageal squamous cell carcinoma (ESCC). By activating epithelial-mesenchymal transition and degrading the extracellular matrix (ECM), miR-212 promotes multiple signaling cascades, cell motility and invasion in ESCC. Berberine may downregulate miR-212 to eventually inhibit cell migration (Chen et al., 2019). In ovarian cancer, miR-21 enhances tumor resistance to chemotherapy. Berberine increases cell sensitivity to cisplatin through the miR-21/PDCD4 axis, by decreasing miR-21 expression and function by enhancing the levels of its target PDCD4, an important tumor suppressor of ovarian cancer (Liu S. et al., 2013). Studies on miRNAs have attracted great attention and contributed to the understanding of the relationship between miRNA and the active components derived from *C. Rhizoma.*

#### The Classical Mechanisms

##### Induction of Apoptosis

The *C. Rhizoma* extract plays its antitumor role in various cancers through induction of apoptosis. In two different human squamous cell carcinoma (SCC-25, KB) cells:, *C. Rhizoma* extract influenced cell differentiation and apoptosis by targeting STAT3, p53, and BRCA1 (Wang et al., 2011). The Bcl-2 family members induce apoptosis (Korsmeyer, 1999). There are two functionally distinct groups of the Bcl-2 family members: anti-apoptotic and pro-apoptotic factors. Bcl-2, an anti-apoptotic protein, mediates the apoptotic pathways and protects cell viability, while Bax, a pro-apoptotic protein, is abundantly and selectively expressed during cell apoptosis, promoting cell death (Oltvai et al., 1993). The pro-apoptotic effects induced by the extracts of *C. Rhizoma* in HCC (HepG2) cells are expressed by downregulating Bcl-2, activating procaspase-3 and procaspase-9, as well as cleaving poly (ADP-ribose) polymerase (PARP) (Auyeung and Ko, 2009). The activity of caspases, particularly Caspase-3, led to a series of morphological and biochemical changes in the apoptosis execution phase (Thornberry and Lazebnik, 1998). As for CRC, the methanol extract of *C. Rhizoma* exerts its anticancer role by activating intracellular death-related pathways, resulting in Caspase-3 activation in human CRC (SNU-C4) cells (Kim et al., 2004). The water extract *C. Rhizoma* influences the viability of melanoma (A2058, UACC257, UACC62, SK-Mel-2, MeWo, M14, Malme-3M) cells, and mouse fibroblast cells. These effects might be partly attributed to apoptosis induction, which may involve the suppression of anti-apoptotic proteins, including BCL2A1, Mcl-1 and Bcl-w, and the activation on multidomain pro-apoptotic proteins, such as Bax and Bak (Xu et al., 2017). *C. Rhizoma* extract induces glioma cell apoptosis. Under treatment with *C. Rhizoma* extract, the expression of proteins associated with apoptosis was altered and involved the reduction of total Caspase-3 and induced cleavage Caspase-3 (Li J. et al., 2017). The water extracts of *C. Rhizoma* exerted apoptotic effects in immortalized human oral keratinocytes through the mitochondrial signaling pathway, represented by mitochondrial cytochrome-C release and Caspase-3 activation (Lee et al., 2006). The root extract of *Coptis japonica* var. *dissecta* (another species of *C. Rhizoma*), functions as an apoptosis inducer by activating Bax-dependent Caspase-3 in human gastric cancer (SNU-668) cells (Park et al., 2005).

Berberine was found to induce apoptosis in many cancer cell lines, including HCC (HepG2) cells (Hyun et al., 2010) and other liver cancer cells (Wang et al., 2010b; Yip and Ho, 2013), human CRC cancer (SW620) cells (Hsu et al., 2007), human gastric cancer (BGC-823) cells (Yi et al., 2015), human cholangiocarcinoma (QBC939) cells (He W. et al., 2012), human pancreatic cancer (MIA-PaCa2, PANC-1) cells (Park et al., 2015), human glioblastoma (87 MG) cells (Palma et al., 2020), and human osteosarcoma (MG-63) cells (Zhu Y. et al., 2014). Apoptosis was mediated by the typical mitochondria-dependent apoptotic signaling pathway, although the activation of the JNK/p38 pathway (Hsu et al., 2007), caspase-independent cell death (Wang et al., 2012), nonsteroidal anti-inflammatory drug (NSAID)-activated gene-1 (NAG-1) activation (Auyeung and Ko, 2009), AKT/mTOR/p70S6/S6 pathway suppression (Yi et al., 2015), ROS generation and cytochrome-C release were also involved in berberine-induced apoptosis (Tillhon et al., 2012). Furthermore, berberine could modulate the MMP-2 and the Bcl-2/Bax signaling pathway to induce cell apoptosis in NSCLC (Li et al., 2018). Meanwhile, berberine induced cell apoptosis in HCC by decreasing phosphorylated AKT and phosphatidylinositol 3-kinase (PI3K) levels in HCC (MHCC97-H and HepG2) cells (Song et al., 2019).

##### Regulation of Signal Transduction

The STAT protein family is an important transcription factor. STATs regulate the active sites of many proteins through phosphorylation and acetylation (Yang and Seto, 2007). The body adapts to the environment and maintains homeostasis through the precise control of the STAT signaling pathway (Yang and Seto, 2007). STAT3 has proven to be an ideal drug target, as sustained activation of STAT3 and strong dependence on STAT3 activity has been identified in approximately 70% of blood and solid tumors, and is involved in tumor proliferation, survival, self-renewal, invasion, and angiogenesis (Calò et al., 2003; Frank, 2007). Aberrant expression or mutation of genes encoding histone acetyltransferase or histone deacetylase (HDAC) enzymes is associated with tumorigenesis (Mai et al., 2005; Leng et al., 2006; Yang and Seto, 2007; Nguyen et al., 2010). *C. Rhizoma* extract acts as an HDAC inhibitor and can inhibit STAT3 phosphorylation by lowering the expression of histone acetyltransferase 3, thus affecting the function and biological characteristics of glioma cells (Li J. et al., 2017). MAPK and PI3K signaling pathways are also involved. *C. Rhizoma* extract regulates MAPK signaling by targeting Raf-1, ERK1/2, p38 and ERK, along with the PI3K signaling pathway by targeting AKT and PTEN in squamous cell carcinoma (KB and SCC-25)cells (Wang et al., 2011).

Adenosine monophosphate-activated protein kinase (AMPK) and PI3K/AKT signaling exert antagonistic activity in cancer development (Tan et al., 2019). Berberine exerts anticancer activity by modulating the AMPK and PI3K/AKT signaling pathways (Huang et al., 2021). For example, berberine exerts anticancer activity on CRC both *in vivo* and *in vitro* by inhibiting PI3K/AKT signaling and by downregulating insulin-like growth factor 2 (IGF2) mRNA-binding protein 3 (IGF2BP3) (Zhang Y. et al., 2020), a member of the oncofetal RNA-binding protein family, and is highly expressed in many different types of cancers (Mancarella et al., 2018). IGF2BP3 activates IGF2 translation, which leads to activate the direct downstream effector PI3K/AKT pathway (Suvasini et al., 2011; Lederer et al., 2014; Zhang Y. et al., 2020). Overexpression of COX-2 correlates with CRC tumorigenesis and leads to cell proliferation and apoptosis inhibition. Furthermore, COX-2 enhances tumor angiogenesis, cell attachment as well as migration and invasion (Wang and Dubois, 2010). In CRC, the JAK2/STAT3 signaling pathway is persistently activated and therefore, resulting in upregulation of the expression of downstream genes, e.g. MMP-2 and MMP-9 (Yang J. et al., 2013; Slattery et al., 2013). Berberine also suppresses CRC cells invasion and metastasis via mediating the expression of COX-2 and PGE2 (Prostaglandin E2, the main product of COX-2 (Wang and Dubois, 2006) to decrease *p*-JAK2/STAT3 signaling and downstream genes MMP-2 and MMP-9 expression (Liu X. et al., 2015). In addition, berberine has a promising antitumor effect in HCC by inhibiting phosphorylation of AKT and PI3K to suppress cell growth, migration, invasion and induce cell apoptosis in HCC (MHCC97-H and HepG2) cells (Song et al., 2019).

The erythropoietin-producing hepatoma (Eph) receptors family is the largest type of receptor tyrosine kinases (RTKs) (Salgia et al., 2018). Two groups exist: Eph A and B. Eph tyrosine kinase receptors and the ephrin ligands are expressed in many types of tumors (Genander and Frisén, 2010). For example, EphB4 is overexpressed in many cancers such as colon, lung, ovarian, prostate, breast, melanoma, endometrial, and pancreatic cancers which is associated with tumor development. (Huang and Li, 2015; Stephenson et al., 2015) (Mertens-Walker et al., 2015; Lv et al., 2016; Merchant et al., 2017). Stimulating Ephrin induces Eph receptor dimerization and autophosphorylation, resulting in activating downstream signaling molecules including PI3K and MAPK (Germain and Eichmann, 2010). The downstream signaling pathways of PI3K and MAPK contribute to cell growth, survival, and migration of different types of cancers (Hu et al., 2014). Therefore, EphB4 is a potential therapeutic target for malignancies (Salgia et al., 2018). Some reports suggested that berberine drives EphB4 inhibitory activity on cancer cell growth (Zhu M. et al., 2019).

##### Cell Cycle Arrest

The *C. Rhizoma* extract inhibits cell proliferation and causes G_2_/M phase arrest in HCC (HepG2) cells to arrest the cell cycle (Auyeung and Ko, 2009). In addition to glioma (BV2, H4, LN299, U251, U87) cells *C. Rhizoma* induces G_2_/M arrest and markedly suppresses cell proliferation, tumor formation, and migration, and prolongs the survival time of glioma cell-bearing mice *in vivo* (Li J. et al., 2017). Cell cycle progression is regulated by cyclins and cyclin-dependent kinases (CDKs) complexes (Vidal and Koff, 2000). *Coptis japonica* Makino (another species of *C. Rhizoma*) extract was also reported to block vascular endothelial growth factor (VEGF)-induced G_0_/G_1_ phase transition by downregulating the expression of cell cycle-regulated proteins, including cyclin D, cyclin E, CDK2, and CDK4 (Kim SH. et al., 2016). *C. Rhizoma* extract regulates the cell cycle in squamous cell carcinoma (KB, SCC-25) cells mediated by CDK4, CDK6, cyclin B1, cyclin E, cyclin D1, and p27 (Wang et al., 2011). Berberine induces G_0_/G_1_ phase arrest in human cholangiocarcinoma (QBC939) cells (He W. et al., 2012), and G_0_/G_1_ phase arrest in CRC and rectal cancer cells (Zhang Y. et al., 2020). Moreover, berberine induces G_2_/M arrest in leukemia (HL-60, WEHI-3) cells by inhibiting cyclin B1 and promoting Wee1 expression (Lin et al., 2006).

##### Inhibition of Cancer Metastasis

Angiogenesis is a significant part in tumor growth and metastasis (Folkman, 1990; Folkman and Shing, 1992), and endothelial migration is an important step in the process of angiogenesis. Cell invasion is another important characteristic of cancer cells. It is initiated by ECM breakdown by MMPs (Nabeshima et al., 2002). *C. Rhizoma* extract significantly suppresses proliferation, migration, and invasion of human umbilical vein endothelial cells (HUVECs) stimulated by VEGF *in vitro* and inhibits VEGF-induced tube formation *in vitro* and micro-vessel sprouting *ex vivo* (Kim SH. et al., 2016). Furthermore, The *Coptis japonica* Makino extract inhibits the expression of MMP-2 and MMP-9.

F-actin, a type of stress fiber, regulates cell motility and polarization. Its reduction inhibits the migration of cancer cells (Chen et al., 2007; Havaki et al., 2007; Smerling et al., 2007; Shao et al., 2018). The Rho/ROCK signaling pathway plays a pivotal role in cancer metastasis (Itoh et al., 1999; Takamura et al., 2001; Leung et al., 2005; Wong et al., 2008). *C. Rhizoma* aqueous extract suppresses the migration and invasion of a highly metastatic HCC (MHCC97-L) cells. It reduces F-actin polymerization and damages to cytoskeleton network (Wang et al., 2010a). Furthermore, *C. Rhizoma* extract affects cell adhesion mediated by E-cadherin and osteopontin in human squamous cell carcinoma cell lines (KB and SCC-25) (Wang et al., 2011). Metadherin (MTDH) functions as an oncogene that facilitates tumor cell invasion and migration, resulting in poor prognosis (Liu Y. et al., 2015). MTDH is highly expressed in many types of cancers, and involved in tumorigenesis and tumor progression in multiple aspects (Liang et al., 2011). In breast cancer, the overexpression of MTDH is closely associated with carcinogenesis, development, metastasis, and chemoresistance (Su et al., 2010). Berberine exerts anticancer activity partially by regulating MTDH expression in breast cancer (Sun et al., 2019b).

miRNAs suppress their target genes and inhibit protein translation, or cause mRNA cleavage (Lu et al., 2005; Volinia et al., 2006; Fabbri et al., 2008; Krol et al., 2010). miR-145, a tumor suppressor gene (Du and Pertsemlidis, 2010; Huang et al., 2012; Dip et al., 2013), is generally downregulated in various types of cancers (Xu et al., 2012; Yang D. et al., 2013). MMP-16 is a target gene of miR-145. Berberine inhibits migration and invasion by promoting miR-145 expression and decreasing MMP-16 expression in the human ovarian cancer (SK-OV-3, 3AO) cells (Li P. et al., 2021). Furthermore, berberine inhibits migration in breast cancer (ZR-75–30) cells by targeting ephrin-B2 (Ma et al., 2017). Ephrin-B2 is a cell-surface protein that contributes to cancer cell survival, invasion, and migration (Lisle et al., 2015; Chen II. et al., 2016; Yang et al., 2016). Some studies have shown that berberine is sensitive to various growth factors (Jabbarzadeh Kaboli et al., 2014). Ephrin-B2 mediates VEGFR2 internalization to inhibit activating the downstream signaling (Sawamiphak et al., 2010; Sentürk et al., 2011). Berberine exerts inhibitory activity in cells with high expression of Ephrin-B2 by downregulating the expression of Ephrin-B2, Syntenin 1, PICK1, MMP-2 and MMP-9, as well as inhibiting the phosphorylation of VEGFR2 and AKT (Ma et al., 2017). Berberine functions through attenuating the expression of COX-2 and PGE2 to decrease JAK2 and STAT3 phosphorylation as well as downstream genes MMP-2 and MMP-9 expression of CRC cells both *in vitro* and *in vivo* (Liu X. et al., 2015). In human A549 lung carcinoma cells, berberine hydrochloride inhibits cell proliferation and promotes cell apoptosis via regulating the MMP-2 and the Bcl-2/Bax signaling pathways (Li et al., 2018).

##### Epigenetic Regulation

Various carcinogenesis-related genetic and epigenetic events have been discovered. An epigenetic modification is a transient, reversible, and heritable change in gene expression with no modification in the DNA sequence and is associated with gene silencing of tumor inhibitors and oncogene activation. Aberrant expression of miRNA is associated with the growth and development of cancers (Calin and Croce, 2006; Pereira et al., 2013). *C. Rhizoma* extract alters miRNA expression profiles and, consequently, hinders cancer development, induces apoptosis, and improves drug sensitivity (Mohammadi et al., 2017). For example, in human liver cancer (MHCC97-L) cells, the aqueous extract of *C. Rhizoma* upregulates miR-21 and miR-23a to exert its anticancer role (Zhu et al., 2011).

DNA methylation most commonly occurs at the cytosine moiety of the CpG dinucleotide and histone, thus affecting the interaction with DNA and chromatin modifying protein. Studies have focused on the role of hypermethylation of tumor suppressor genes and global hypomethylation of oncogenes (Puneet et al., 2018). There are two forms of human CpG: one is dispersed in genomic DNA and the other is highly clustered to form CpG islands. When a tumor occurs, the degree of CpG unmethylation increases outside CpG islands, while those located within CpG islands are highly methylated, causing the overall decrease of methylation level of the genome and CpG islands (Crawford et al., 2018). The DNMT family related to DNA methylation includes DNMT1, DNMT3A, and DNMT3B (Jackson et al., 2004; Smith and Meissner, 2013). In multiple myeloma (U266) cells, berberine inhibits the expression of DNMT1 and DNMT3B to alter the CpG methylation of *p53*, which affects the mRNA levels of apoptosis-related proteins and thus, induces cell apoptosis and cell cycle arrest (Qing et al., 2014; Liu et al., 2019). In CRC, berberine increases the expression of DNMT1, DNMT3A, DNMT3B and miR-152, miR-429, miR-29a (Huang C. et al., 2017). Additionally, histones are associated with tumorigenesis and development. Histones protect DNA structure and genetic information and regulate gene expression. Berberine downregulates histone deacetylases in lung cancer (A549) cells, resulting in a decrease in the expression of mRNA and protein of the MMP-2 and MMP-9, inhibiting cell migration and invasion (Kalaiarasi et al., 2016).

### Other Alkaloids

In recent years, the pharmacological effects of other alkaloids derived from *C. Rhizoma* have been explored gradually, including coptisine, columbamine, palmatine, jatrorrhizine, magnoflorine, oxyberberine, epiberberine, oxyepiberberine, dihydroberberine, berberrubine, and worenine.

**Coptisine** exerts strong antiproliferative activities in pancreatic cancer (MiaPaCa-2, Panc-1) cells (Hara et al., 2005). It also exerts active anticancer effect on hepatoma (HepG2, Hep3B, SK-Hep1, and PLC/PRF/5), leukemia (K562, U937, P3H1, and Raji) and osteosarcoma (MG63) cells (Lin et al., 2004; Yu et al., 2014). An *in vivo* study using HCT-116 xenograft mouse mode shows its anticancer effects in breast cancer therapy (Luo et al., 2014; Huang T. et al., 2017). Briefly, the corresponding mechanisms of action involve cell cycles arrest, apoptosis induction, and metastasis inhibition. Coptisine effectively induces G_0_/G_1_ phase arrest in CRC (HCT-116 and FHC) cells and pancreatic carcinoma (PANC-1) cells (Huang T. et al., 2017; Zhang Y. et al., 2020). It also causes G_0_/G_1_ phase arrest by downregulating the expression of CDK4 and cyclin D1 in a xenografted mouse model (Yu et al., 2014). Furthermore, coptisine can also induce cell apoptosis. In HCT-116 cells, coptisine induces G_1_ phase arrest and caspase-dependent/independent apoptosis through suppressing the levels of PI3K and AKT, mediating Bcl-2 family to the mitochondria-associated apoptotic pathways (Huang T. et al., 2017; Han et al., 2018). Finally, coptisine plays an important role in inhibiting tumor migration and invasion. In osteosarcoma, it decreases the mRNA levels of some tumor angiogenic genes, such as VE-cadherin and integrin β3 and suppresses STAT3 phosphorylation so as to potently impede cell migration and invasion (Yu et al., 2014). In human breast cancer cells with high metastasis potential, coptisine exerts antimetastatic function by downregulation of MMP-9 in combination with increases in tissue inhibitor of metalloproteinase 1 (TIMP-1) (Luo et al., 2014). Furthermore, coptisine also triggers autophagy in Hep3B cells via downregulating the phosphorylation of mTOR and ULK-1 as well as its upstream pathway PI3K/AKT and upregulation of ROS-mediated mitochondrial dysfunction (Kim et al., 2021).

**Columbamine** exerts antiproliferative, anti-metastasis, anti-vasculogenic, and cytotoxic effects in various cancer types, including human CRC, HCC, and metastatic osteosarcoma (Zhang et al., 2014; Lei et al., 2019). Columbamine significantly decreases tumor volumes in HCT116 or SMMC7721 xenograft mouse model, as well as induces cell apoptosis and G_2_/M phase arrest via Wnt/β-catenin, MAPK, CDK6, STAT3 signaling pathways in *in vitro* models (Lin et al., 2019). Simultaneously, it downregulates MMP-2 expression and reduces cell migration and invasion in HCT116, LoVo, and U2OS cells (Wang et al., 2012).

**Palmatine** has been reported to have anticancer effects in CRC, HCC, pancreatic cancer, oral squamous cell carcinoma, breast cancer, ovarian cancer, and prostate cancer both *in vitro* and *in vivo* (Long et al., 2019; Qi et al., 2019). It induces antiproliferative effects, anti-inflammatory effects, G_2_/M phase arrest, and cell apoptosis via mitochondrial pathways by targeting cytokines and aurora kinase A (AURKA) in CRC cells (Ma et al., 2016; Liu et al., 2020e). Particularly, it is identified to exert anti-*Helicobacter pylori* activity, which is a trigger for gastritis and gastric cancer, showing a great potential in prevention and therapy of *Helicobacter pylori*-induced gastric cancer (Jung et al., 2014).

**Jatrorrhizine** has been reported to have an inhibitory effect on HCC, breast cancer, and melanoma. For breast cancer, it induces apoptosis through caspase-mediated mitochondrial pathway and potentially inhibits cell proliferation and metastasis by repressing Traf2 and Nck interacting serine protein kinase (TNIK) and epithelial-mesenchymal transition (EMT) via Wnt/β-catenin signaling *in vitro* and in 4T1 tumor-bearing mice (Sun et al., 2019a). Similarly, jatrorrhizine suppresses proliferation of metastatic melanoma cells by inducing G_0_/G_1_ phase arrest by enhancing the expression of *p21* and *p27* genes, and hinders human melanoma C8161 cell-mediated neovascularization *in vitro* and *in vivo*, which is accompanied by downregulation of VE-cadherin (Liu R. et al., 2013).

**Magnoflorine** suppresses cell proliferation of human gastric cancer by generating ROS, especially in SGC7901 cells and the xenograft tumor, by inducing autophagy via LC3B-II upregulation, G_2_/M phase arrest via p27 and p21 upregulation, and cell death via caspase activation (Sun XL. et al., 2020). Moreover, for breast cancer, it improves cell sensitivity to doxorubicin through inducing autophagy and apoptosis by elevating LC3-II and activating Caspase-3 via MAPK signaling pathway (Wei T. et al., 2020).

**Oxyberberine** can significantly alleviate DSS-induced colitis in mice and the effect is superior to berberine. Furthermore, oxyberberine can improve the colonic inflammatory reaction and intestinal epithelial barrier function. It inhibits the inflammatory signaling pathway via downregulation of inflammatory cytokines and the expression of TLR4 and MyD88, which inhibits the phosphorylated IκBα, and the translocation of NF-κB-p65 from the cytoplasm to the nucleus. In addition, oxyberberine markedly modulates DSS-induced intestinal dysbiosis and restores the balance of intestinal microbiota (Li C. et al., 2020).

In MKN-45-related gastric cancer, which harbors wild type p53, **epiberberine** shows great potential in inhibiting cell growth by inducing ROS, mitochondrial apoptosis and cell cycle arrest *in vitro* and inhibiting tumor growth *in vivo* via the p53/Bax pathway, and also inhibiting cell growth in the human gastric cancer (HGC-27) cells, which harbors mutated p53 (Zhai et al., 2020). Lysine-specific demethylase 1 (LSD1) is considered a critical target in many cancer treatments (Fiskus et al., 2014; Hong et al., 2018; Hu et al., 2019). Epiberberine acts as a LSD1 inhibitor in THP-1 and HL-60 cells (Li ZR. et al., 2020). Meanwhile, epiberberine also inhibits telomerase by disrupting telomere maintenance and capping to induce cell apoptosis (Liu L. et al., 2020). Furthermore, epiberberine induces low expression of Bcl-2 and the X-linked inhibitor of apoptosis protein (XIAP), the high expression of Bax, p53 and release of cytochrome C, and the activation of Caspase-3 to promote gastric cancer cell apoptosis (Liu L. et al., 2020). **Oxyepiberberine** has been reported to inhibit proliferation of CRC LS-1034 cells both *in vitro* and *in vivo*, accompanied by inducing apoptosis and inhibiting migration and tubulin polymerization (Ning et al., 2021). In addition, it inhibits cancer lung metastasis, impeding TGF-β1-induced EMT in cancer cells, and preferentially interfering with the Smad3 promoter activity in EMT (Liu et al., 2020f).

**Dihydroberberine**, an alkaloid of isoquinoline with various bioactivities, was identified in many plants including *C. Rhizoma*. Dihydroberberine has a therapeutic effect on UC induced by DSS *in vivo* by suppressing the immune-inflammatory response. It may decrease IL-6, IL-1β, IL-17, IFN-γ, TNF-α, and IgA by suppressing TLR4/MyD88/NF-κB signaling pathway to alleviate colonic immune-inflammation (Li C. et al., 2021). Meanwhile, it ameliorates inflammation by improving gut barrier function by upregulating the levels of claudin-1, occludin, junctional adhesion molecules A (JAM-A), and mucins (Li G. et al., 2021).

**Berberrubine,** a specific poison of topoisomerase II, has a potent antitumor activity by inducing DNA cleavage *in vitro* and exerts remarkable upgradation resistance to CRC associated with downregulation of topoisomerase IIα *in vivo* (Kang and Chung, 2002).

**Worenine** inhibits cell viability and proliferation, as well as induces G_2_/M phase arrest by balancing the Warburg effect via HIF-1α signaling in CRC cells (Ji L. J. et al., 2021).

### The Derivatives

The chemical structures of compounds of *C. Rhizoma* and some active derivatives have been identified, as shown in Figure 1. A series of compounds have been synthesized that exhibit differences relative lipophilicity due to the size of the substituent achieved by extending the alkyl side chain. This structure modification increases cytotoxic activity, and some derivatives (**8-phenyl-berberine** and **8-n-butyl-berberine**) show better selectivity for breast cancer cells as well. **13-n-butyl-berberine** and **13-n-hepta-berberine** increase cytotoxic activity and are very effective against lung cancer (Iwasa et al., 2001). A study showed that 13-methylberberine and 13-ethylberberine could be used as potential immunotherapeutic compounds to induce IL-12, and combined with an iNOS inhibitor has potential value in cancer treatment (Lee et al., 2003). **8-cetylcoptisine**, a new derivative of coptisine, exerts anticancer activity by inducing mitochondria-related apoptosis and G_0_/G_1_ phase arrest. 8-cetylcoptisine significantly delays tumor development in NSCLC (A549) cells-bearing mice, which is much stronger than coptisine. In addition to A549 cells, 8-cetylcoptisine also inhibits cell viability of BGC-823, HepG2, HCT-116 and MDA-MB-231 cells (Han et al., 2019).

**Demethylenetetrahydroberberine** (DMTHB) is a new derivative of berberine that can improve the symptoms of nonalcoholic fatty liver disease (NAFLD) by repressing the NLRP3 inflammasome and oxidative stress in mice. DMTHB targets NLRP3 inflammasome and TLR4/NF-κB signaling to suppress inflammatory response and inhibits CYP2E1 and C/EBP homologous protein (Kumar et al.), therefore activating transcription factor 4 (ATF4) to repress the over-expression of ROS and endoplasmic reticulum stress (Zhang Y. et al., 2021).

### Others

Some flavonoids, lignans, and other natural compounds are derived from *C. Rhizoma* and exists in many other botanical drugs. **Limonin** has potential effects against CRC, HCC, breast cancer, panchromatic islet cancer, meningioma, lung cancer, leukemia, and cervical carcinoma (Shimizu et al., 2015; Tang et al., 2019; Chen et al., 2021). The critical targets of action of limonin were described in detail in a recent review, including NAD(*p*)H quinone oxidoreductase 1 (NQO1), Yes-associated protein 1 (YAP1), NF-κB, p53, Wnt, and STAT3 (Fan S. et al., 2019). Limonin also overcomes MDR of antitumor agents (e.g., DOX) by inhibiting the activity of P-glycoprotein (P-gp) (El-Readi et al., 2010; Fan S. et al., 2019).

**Rhamnetin** induces cell death by provoking apoptosis, suppresses cell migration by inhibiting EMT, reverses MDR by reducing P-gp and breast cancer resistance protein (BCRP) expression, and enhances the antitumor effects of sorafenib, etoposide, paclitaxel, irradiation via the miR-34a/Notch-1 and miR-148a/PXR axis in HCC (Jia et al., 2016; Li B. et al., 2021), breast cancer (Lan et al., 2019), and NSCLC (Kang et al., 2013).

Growing evidence proved that **Wogonin** (Ming et al., 2020), **Pinoresinol** (Sain et al., 2021)**, Secoisolariciresionol** (Ozgocmen et al., 2021)**,** and **Vanillic acid** (Gong et al., 2019b) have a high potential to tackle gastrointestinal cancer through regulating cell cycle arrest, cellular senescence, autophagy, as well as inhibiting angiogenesis via multiple mechanisms.

## OMICS Studies

Undoubtedly, traditional approaches for biological analysis require extensive resources to evaluate the increasing information derived from scale data sets in cancer research and drug discovery. With the rapid development of science and technology, omics offers a multidimensional perspective in a convenient approach to anticancer drug discovery, and includes genomics, proteomics, metabolomics, and microbiomics.

### Genomics

Genome-wide expression profiling of cancer cells treated with *C. Rhizoma* and its components have been investigated using cDNA microarray, oligonucleotide microarray, and RNA sequencing. These studies reveal the anticancer effects of *C. Rhizoma* by the regulation of altered gene expression profiles. A genome-wide biological response fingerprinting (BioReF) study showed that most genes are enriched in the terpenoid backbone biosynthesis pathway in HCC (HepG2) cells, which is remarkably influenced by treatment with *C. Rhizoma* originating from specific growing regions, along with six downregulated gene sets involved in the mevalonate pathway (Feng et al., 2018). To further explore this relationship, a total of 27 differentially expressed genes (DEGs) selected among 12,600 genes were identified to be involved in the regulation of signal transduction, cell metabolism, and cell invasion (MMP-14 and PAK1), that contribute to the anticancer effects of *C. Rhizoma*, as well as 3,726 genes that were downregulated and 3,642 genes upregulated by berberine in pancreatic cancer cells (Hara et al., 2005; Liu et al., 2020b). In addition, bioinformatics is an indispensable tool in omics analysis, which reveal the underlying mechanisms of the anticancer activity of *C. Rhizoma*. For example, the comparison of gene expression and survival analysis demonstrates that MYC is overexpressed in colorectal tumors and is highly associated with poor overall survival. *C. Rhizoma* was identified to target MYC using the TCMSP (https://tcmsp-e.com/) (Ru et al., 2014) and TCM-MESH (http://mesh.tcm.microbioinformatics.org/) (Zhang et al., 2017) databases, respectively (Dong et al., 2019). Moreover, 56 upregulated and 8 downregulated genes of berberine-treated HCC (HepG2) cells were identified and enriched in cell cycle, cell apoptosis, and transcription (Hu et al., 2018). Berberine could suppress cell proliferation and induce apoptosis by upregulating 1,960 genes and downregulating 4,837 genes, that are involved in cellular, metabolic, and single-organism processes in gastric cancer (SGC-7901) cells (Yang et al., 2018). A total of 2,706 and 3,397 DEGs involved in the regulation of cell apoptosis, cell cycle, and cell migration are regulated by berberine in breast cancer (MDA-MB-231 and MCF-7) cells. (Wen et al., 2013). Collectively, the application of genomics in anticancer research on *C. Rhizoma* is still in its infancy. In particular, genomic approaches help to accelerate high-throughput screening to identify the target genes of *C. Rhizoma*. However, a transcriptomic analysis has rarely been employed to reveal the anticancer action of *C. Rhizoma* and its components.

### Proteomics

Proteomics analysis, the application of gel electrophoresis combined with mass spectrometry (Chou et al.), has been applied to reveal the potential therapeutic targets of berberine in CRC, HCC, breast cancer, cervix adenocarcinoma, and melanoma. Proteomic data demonstrated differentially expressed proteins (DEPs) among 5,130 or 8,051 identified proteins on berberine-treated CRC DLD-1 (675 proteins), HCT-116 (865 proteins), Caco-2 (503 proteins), and LOVO (277 proteins) cells, respectively. Among these, a total of 54 DEPs (22 upregulated and 32 downregulated proteins) overlapped in DLD1 and HCT116 cells, as well as 83 DEPs (the most downregulated proteins) overlapped in Caco-2 and LOVO cells were mainly involved in specific pathways, including calcium mobilization (LAT2/NTAL/LAB), metabolism of fat-soluble vitamins (LDLR and VKORC1), mitochondrial protein synthesis (GTPase ERAL1, MRPL11, and MRPL 48), tricarboxylic acid cycle (TCA) (citrate synthase, CS), and respiratory electron transport pathway (NDUFS2 and COX7A2L) (Tong et al., 2020; Li P. et al., 2021). Moreover, a total of 96 and 22 DEPs screened from 1,800 identified proteins showed altered protein expression and thiol reactivity on berberine-treated breast cancer (MCF-7) cells, respectively, which are attributable to gene regulation (HDAC1), protein folding (HSP27 and PPIA), signal transduction (KCIP-1 and NRG2), and metabolism (ENO1 and TPI1) (Chou et al., 2012). In berberine-treated cervix adenocarcinoma HeLa cells, a total of 51 DEPs screened among more than 700 proteins were strongly associated with cellular metabolic process (PDHB and MAPK13), cellular component organization (HSPA8 and VIM), and cell apoptosis and proliferation (ANXA5 and PHB) (Lu et al., 2012). A total of 23 DEPs (3 upregulated and 20 downregulated proteins) identified from approximately 806 proteins were associated with cell death (Annexin A1, Ezrin, and Septin-8), molecular chaperone (TCP-1 and Ferritin), and metabolism (Elongation Factor Tu and UQC. RHIZOMAC1) in melanoma (B16F10) cells treated with berberine (Kim J.-H. et al., 2016). In addition, a total of 8 DEPs (3 upregulated and 5 downregulated proteins) from berberine-treated HCC (HepG2) cells were associated with cell proliferation (MAPK4), cell metabolism, cell cycle, and DNA damage response (Tan et al., 2006). Accumulating evidence has shown that proteomic approaches could be helpful to investigate the mechanism of anticancer activity of berberine, but few analyses of proteomics, metabolomics, and microbiomics have been performed to identify the therapeutic targets of *C. Rhizoma* extract and other bioactive components in cancer cells. Further experimental verification, such as Western blotting, immunoprecipitation, and immunofluorescence, should be performed to confirm the potential targets identified from high-throughput proteomic data.

### Metabolomics

With the advancement of technologies, newly developed targeted metabolomics approaches combined with MS have been applied to accurately identify berberine-induced changes in metabolism in CRC, HCC, pancreatic cancer, and prostate cancer. In berberine-treated pancreatic cancer cells, the metabolism of 78 differential metabolites (DMs) was significantly changed at the phenotype level. These DMs included metabolism of some amino acids and nucleotides, upregulated energetic metabolism (glycolysis and glutamine)-associated metabolites and downregulated TCA cycle-associated metabolites (citrate), which interfere with mitochondrial structure and dysregulate energy metabolism (Eladl et al., 2020). Moreover, a total of 14 DMs (3 upregulated metabolites and 11 downregulated metabolites) in prostate cancer cells and 30 DMs (16 upregulated metabolites and 14 downregulated metabolites) in blood are closely associated with the metabolism of phenylalanine, d-arginine, d-ornithine, tyrosine, and the metabolism of arachidonic acid, glycerophospholipid, linoleic acid, purine, sphingolipid, retinol, the TCA cycle, arginine and proline (e.g., arachidonic acid, cholines, citric acid, eicosapentaenoic acid, prostaglandin A1/A2, thromboxane, and uric acid) (Li X. et al., 2017). Recently, Feng et al. from the University of Hong Kong reported that a total of 53 metabolites from tumors and 25 metabolites from HCC (MHCC97L) cells have been identified that could mediate berberine in the regulation of the glucose–alanine cycle (e.g., sugar, fatty acid, amino acid, and organic acid) (Guo et al., 2020).

Phylogenetic Investigation of Communities by Reconstruction of Unobserved States (PICRSt) analysis was used to predict the metabolic functions for berberine treatment based on 16S rRNA. The results indicated that berberine intervention along with the reduction of intestinal tumor development could improve metabolic abnormalities by reversing glucose metabolism (glycolysis/gluconeogenesis, fructose and mannose, and galactose), amino acid-related metabolism (lysine degradation), and lipid metabolism (fatty acid biosynthesis and arachidonic acid). *In vivo* animal models confirmed the above mechanisms by analyzing a total of 8 DMs from the detected 44 of fecal metabolites regulated by berberine in the azoxymethane/DSS-induced CRC mouse model (Chen et al., 2020b). To uncover the interactions of DMs and further investigate the underlying mechanisms of action of berberine against prostate cancer, bioinformatics analysis was performed to identify the functions of metabolites using Ingenuity Pathway Analysis (IPA) software (Li X. et al., 2017). Hence, further in-depth study on large-scale biological data sets is required using multiple bioinformatics approaches to provide a more holistic view, which also include the crosstalk between metabolites and microbiota.

### Microbiomics

The microbiota exists in all multicellular organisms and plays an important role in carcinogenesis. Growing attention has been focused on the gut microbiota and its impact on cancer progression (Elinav et al., 2019; Jaye et al., 2021; Matson et al., 2021). Microbiomics is a feasible approach to detect alterations in the gut microbiota including bacteria, fungi, archaea, and viruses using 16S rRNA gene sequencing, whole metagenome sequencing (WMS), pyrosequencing sequencing, gene chip, and fluorescence *in situ* hybridization (Petrosino et al., 2009). Accumulating evidence suggests that berberine exerts anticancer effects by improving intestinal dysbacteriosis in CRC and breast cancer. Below we will examine the so-called hidden organ (gut microbiota) effects induced by berberine.

In fecal or mucosal samples, berberine treatment greatly rescues colorectal tumorigenesis by improving the imbalance of the gut microbiota and modulating the tumor microenvironment by decreasing the richness of microbiota community and relative abundance of Actinobacteria and Verrucomicrbia at the phylum level, as well as suppressing pathogenic species (e.g., *F. nucleatum*, *f_*Erysipelotrichaceae and *Alistipes*) and elevating some bacteria producing short-chain fatty acid (SCFA) (*Alloprevotella*, *Flavonifractor* and *Oscillibacter*) at the genus level in AOM/DSS-induced CRC mouse model (Yu et al., 2015; Chen et al., 2020b). Interestingly, exercise therapy combined with berberine treatment produces the lowest effects on species richness and the highest relative abundance of bacteroidaceae to achieve a synergistic anticancer effect, compared with negative control and single administration in 4T1 breast cancer-bearing mice (Ma et al., 2020). Strong evidence supports that dysbiosis of gut microbiota involved in inflammatory and immune response is directly correlated with the development and concurrence of CRC (Alexander et al., 2017), thus, most studies for the anticancer effects of berberine have concentrated on the gut microbiota and inflammation in CRC. Further studies should also be performed to explore microbiota in the other cancer models for anticancer effects of *C. Rhizoma* and its components.

## Novel Drug Delivery Systems

By changing drug delivery systems, a novel mode of drug administration with improved physical and/or chemical properties and bioavailability could be obtained, and the expected activity or efficacy could also be improved. Systemic/targeted drug delivery systems, including liposomes, nanoparticles, and hydrogels, could decrease side effects, toxicity, and the frequency of administration, also overcome MDR, improve symptoms and the survival rates in cancer treatment.

To improve bioavailability and optimize berberine immunoregulatory effects in a specific part of the gastrointestinal tract, a micro- and nanoencapsulated hybrid delivery system, targeted colon by oral administration, was established by encapsulation of poly nanoparticles loaded with berberine into a pH-sensitive Eudragit FS30D matrix pre-entrapped with berberine to form a hybrid microparticle. The nanoparticles are able to improve release of berberine subsequent to their intestinal absorption. Treatment with this novel system results in a more effective improvement than free berberine in acutely and chronically induced colitis by DSS. The improved efficacy is accompanied by a decrease in colon inflammation (Zhang L. et al., 2021).

Berberine combined with carboxylmethyl chitosan by arylboronic ester becomes a new oxidation-responsive nanoprodrug. It is responsive ROS and effectively delivers drugs to inflamed tissues (Lamprecht et al., 2001; Wilson et al., 2010; Zhang et al., 2016). The combination of carboxylmethyl chitosan modified with phenylboronic esters and berberine can significantly improve the symptoms of colitis and colon damage by regulating IL-6 expression and remodeling the intestinal microbiota (Zhao L. et al., 2021).

Synthesized silver nanoparticles (AgNPs) have attracted great attention because of their excellent characteristics, such as easy surface modification and synthesis, and superior biocompatibility (Gupta and Gupta, 2017). In a study, researchers found that AgNPs in combination with an aqueous extract of *C. Rhizoma* inhibited proliferation, migration, and invasion in NSCLC (A549) cells, and induced apoptosis likely involving the mitochondria-mediated pathway (Pei et al., 2019). To improve the poor oral bioavailability of berberine, new formulations of nano emulsion protocols have been explored and have achieved better bioavailability, efficacy, and pharmacological activity via the P-gp efflux system (Kumar et al., 2015; Liu et al., 2016). When berberine nanoemulsions are combined with photodynamic therapy, this novel treatment modality induced significant phototoxicity in the cervical carcinoma (Caski) cells and spontaneously immortalized non-tumorigenic human skin HaCaT keratinocytes, exhibiting a great potential for working as photosensitizing agents in cervical carcinoma treatment (Floriano et al., 2021).

Folate acid-modified chitosan nanoparticles loaded berberine hydrochloride are effective in regulating cell proliferation, migration, and apoptosis by improving its hydrophobic properties, poor stability, and bioavailability in nasopharyngeal carcinoma (CNE-1) cells both *in vivo* and *in vitro* (Wang et al., 2018). Although coptisine was reported to show positive anticancer effects in some studies (Lin et al., 2004; Hara et al., 2005; Luo et al., 2014; Yu et al., 2014; Huang T. et al., 2017; Han et al., 2018; Zhang YL. et al., 2020; Kim et al., 2021), its poor absorption and low bioavailability were still obstacles for its application. Therefore, nano strategies, microrods and salt formulation could also be employed to promote the intestinal dissolution (Wu et al., 2019).

## Combination Therapy

### Combination With Chemotherapeutic Drugs

In most cases, monotherapy is not as effective as expected, and is always required excessive dosages and accompanied with significant side effects. Combination of anticancer drugs with *C. Rhizoma* extract or active ingredients could improve MDR, exert synergistic effects, and reduce side effects, which contributes to improving clinical outcomes in chemotherapy and is regarded as a beneficial treatment for cancers. There are many studies that support the potential of *C. Rhizoma* as an adjuvant agent in chemotherapy.

#### Improvement of Multidrug Resistance

MDR is a critical limitation of chemotherapy, and its corresponding mechanisms have been extensively explored. The potential mechanisms of MDR include alterations in the expression of the ABC transporter family members, apoptosis induction, autophagy induction, cancer stem cell regulation, miRNA regulation, hypoxia induction, DNA damage and repair, and epigenetic regulation (Wu et al., 2014). The mechanisms involving *C. Rhizoma* that improve MDR are described below.

P-gp transporter and multidrug resistance protein 1 (MRP1) are active members of ABC transporters in the gastrointestinal system. Overexpression of P-gp in CRC patients leads to drug efflux and drug resistance (Lee et al., 2018). In the human CRC (HCT15) cells and the human MDR uterine sarcoma (MES-SA/DX5) cells, 8-oxocoptisine, which is obtained from the rhizome of *Coptis japonica* Makino, shows significant efficacy as a P-gp inhibitor. Its inhibitory activity on P-gp is similar to that of verapamil (Min et al., 2006). Thymidylate synthase (TS) is reported highly expressed in CRC patients and is associated with overall survival (Popat et al., 2004). *C. Rhizoma* extract significantly reduced the IC_50_ value of fluorouracil (5-FU) in human CRC (HCT116) cells, and could act as a potential adjuvant agent against 5-FU-resistant CRCs by attenuating the expression of *TS* gene (Kang et al., 2021). In addition, *C. Rhizoma* extract improves the sensitivity of HeLa cells to paclitaxel or 5-FU, presumably via the inhibition of P-gp function (Takara et al., 2005). TNF-related apoptosis inducing ligand (TRAIL) is considered a potential adjuvant in cancer treatment (Johnstone et al., 2008). However, some cancer cells appear to resistant to TRAIL-induced apoptosis (Stegehuis et al., 2010). TRAIL resistance was reported to be reduced by the combination of TRAIL and *C. Rhizoma* aqueous extract in TRAIL-resistant A549 cells (Chiang et al., 2018). As a potential anticancer agent for NSCLC, *C. Rhizoma* extract and its major constituent berberine repress ROS production, ameliorate MDR, and enhance the inhibitory effects of chemotherapeutic agents in A549 cells (He C. et al., 2012). The EGFR tyrosine kinase inhibitor (TKI), gefitinib, is used in the treatment of NSCLC with EGFR mutations (Rawluk and Waller, 2018). *C. Rhizoma* extracts suppress EGFR/AKT signaling and the expression of overexpressed antiapoptotic proteins, Mcl-1 and Bcl-2 in gefitinib-resistant NSCLC cells (Kim et al., 2020).

Long term treatment of DOX can induce MDR by transforming N1 into N2 neutrophil phenotypes via upregulation of CD133, CD309, and PD-L1 expression in HL-60 cells. Treatment using the combination of berberine and DOX can maintain the N1 neutrophil polarization and stimulate immune checkpoints to reverse MDR by downregulating CD133, CD309, PD-1, and PD-L1 expression in HCC allograft model (Zhang et al., 2019).

#### Synergistic Effects

Drug combinations often exhibit synergistic effects, which can be achieved by a combination of two or more drugs, making combined treatment much more effective than monotherapy. Aiming at the same target, increasing the effect of the former drug or improving the microenvironment in which the drug acts, all may effectively increase clinical efficacy. The primary metabolites of the *C. Rhizoma* extract, known as natural deep eutectic solvents, improves the pharmacokinetics of orally administered berberine (Zhao J. et al., 2021).

*C. Rhizoma* extract and 5-FU act on the same target. Cotreatment with *C. Rhizoma* extract and 5-FU significantly augments G_0_/G_1_ phase arrest, especially in 5-FU-resistant CRC. The potential mechanisms might be associated with modulation of TS expression (Kang et al., 2021). Additionally, cotreatment with *C. Rhizoma* extract and estrogen receptor (ER) antagonists increase the anticancer effect. A majority of breast cancer patients are ER positive and the disease progresses in the presence of high levels of estrogen (Jordan, 2008). ER antagonists, such as tamoxifen and fulvestrant, have been widely used in the treatment of ER-positive breast cancer, but the drug efficacy and resistance remain a concern (Shou et al., 2004; Abukhdeir et al., 2008; Jordan, 2008; Lee et al., 2008). Combined treatment of *C. Rhizoma* extracts or berberine with tamoxifen were found to enhance the inhibitory effects on ER-positive breast cancer (MCF-7) cells via downregulating the expression of EGFR, HER2, bcl-2 and COX-2 and upregulating IFN-β and p21 (Liu et al., 2009).

The ErbB family is a type of TK receptors, with high expression in breast cancer patients (Scott et al., 1991; Witters et al., 2007). EGFR and HER2, two members of the ErbB family, antagonize the anticancer effects of tamoxifen and induce drug resistance via activating ER and co-regulatory proteins. Bcl-2 inhibitors induce apoptosis and improve cell sensitivity to other therapies. It would be beneficial to block the ErbB family and the Bcl-2 family simultaneously (Witters et al., 2007). High expression level of COX-2 also reduces the inhibitory effects of tamoxifen on breast cancer cell growth (Tari et al., 2005). The upregulation of IFN- β induces the antiproliferative effect of *C. Rhizoma* extract (Buzzi et al., 1992). p21, which acts as a potent CDK inhibitor, mediates the p53-dependent G_0_/G_1_ and S phase arrest. The loss of p21 expression is associated with resistance to tamoxifen in breast cancers (Abukhdeir et al., 2008).

The combination of berberine and PEGylated liposomal doxorubicin (PEG-lip-DOX) suppresses tumor growth, which is much more effective than that achieved by monotherapy with berberine or PEG-lip-DOX treatment alone. This combination also exhibits antiproliferative activity on Meth A murine sarcoma cells (Yahuafai et al., 2018). Berberine improves the sensitivity to cisplatin of breast cancer (MCF-7) cells. Pro-apoptotic Capase-3 and cleaved Caspase-3 and Caspase-9 were induced and Bcl-2 was downregulated after cotreatment of berberine and cisplatin (Zhao et al., 2016). Proliferating cell nuclear antigen (PCNA) is a DNA sliding clamp required for DNA pol δ to replicate DNA and is crucial in DNA repair (Zhu Q. et al., 2014). Berberine restrains the expression of cellular PCNA and increases DNA damage induced by cisplatin. A decrease in the cellular DNA repair ability may result in cell sensitizing to genotoxic cisplatin (Zhao et al., 2016).

Berberine enhances the expected maximum antitumor activity of 17-AAG (the Hsp90 inhibitor) and SAHA (the HDAC inhibitor) alone in CRC (SW480) cells. The combination of the three appear the most effect (Li J. et al., 2021). Magnoflorine improves the cell sensitivity to doxorubicin through inducing autophagy and apoptosis by elevating LC3-II and activating Caspase-3 via MAPK pathway in breast cancer. This combination suppresses migration and invasion, induces G_2_/M arrest, apoptosis and autophagy (Wei T. et al., 2020).

#### Reduced Side Effects and Enhanced Efficacy

Cachexia is a complex syndrome that often occurs in advanced cancer patients, with the main symptoms including anorexia, weight loss, and loss of adipose tissue and skeletal muscle (Evans et al., 2008). IL-6 plays an important role in cancer-induced cachexia. It has been demonstrated that downregulation of IL-6 levels would improve cachexia or malnutrition in patients (Strassmann et al., 1992; Fujita et al., 1996; Oka et al., 1996; Cahlin et al., 2000). Botanical drugs might represent an effective approach to cancer-related cachexia as well as in adjuvant therapy (Park et al., 2019). *C. Rhizoma* water extract has anti-inflammatory effects, exerting an anticachectic effect in esophageal cancer (YES-2) cells-bearing mice (Iizuka et al., 2000). The inhibitory effects on the production of pro-inflammatory cytokines by *C. Rhizoma* water extract and berberine were also found to be syngeneic in CRC cells-bearing mice (Iizuka et al., 2002). Furthermore, some adverse effects can also be alleviated by *C. Rhizoma*. Oral mucositis (OM) is a common complication of radiotherapy and chemotherapy which brings pain to head and neck cancer patients due to ROS generation. Hangeshashinto (HST), a Japanese traditional medicine, contains *C. Rhizoma* as one of its components. *C. Rhizoma* and berberine alleviate OM by reducing ROS levels (Matsumoto et al., 2015).

### Combinations With Other Botanical Drugs

*C. Rhizoma* is often formulated in combination with other botanical drugs. These types of herbal formulas play an important role in cancer treatment. They exert special synergistic mechanisms and therapeutic characteristics. There are some common classical prescriptions, such as Zuojinwan, San-Huang-Xie-Xin Decoction, Chingwaysan, and Gegen Qinlian Decoction.

Zuojinwan is a representative Chinese herbal formula, consisting of *C. Rhizoma* and *Euodiae Fructus* at a ratio of 6:1 (w/w). In human gastric carcinoma (SGC-7901) cells, water extracts of *C. Rhizoma* and *Evodiae Fructus* inhibit proliferation and induce apoptosis. The optimal proportion is also 6:1 (w/w) in treating cancer (Peng et al., 2011). Meanwhile, water extracts of *C. Rhizoma* and *Evodiae* Fructus significantly inhibit aberrant crypt foci formation in model animals. A clinical therapeutic effect is observed in CRC, in part due to inhibition of proliferation of the middle and distal crypts and the promotion of apoptosis (Dong et al., 2010). The anticancer activity of Zuojinwan is superior to that of *C. Rhizoma* or *Euodiae* Fructus used alone. An obvious synergistic effect could be associated with gene expression and the activities of tumor markers in the serum (Wang et al., 2009). Berberine and evodiamine are the two main bioactive compounds in Zuojinwan, both inhibit AP-1 and/or NF-κB activities and suppress anchorage-independent growth of HCC (HepG2) cells (Chao et al., 2011). Another study showed that *c-myc* plays a critical role in its cytotoxic effect (Chou et al., 2017). The combination of berberine and evodiamine in human HCC (SMMC-7721) cells significantly enhanced apoptosis and showed the highest inhibitory effect when compared with either used individually (Wang et al., 2008). Furthermore, the combination of berberine and evodiamine also exerts synergistic anticancer effect on P-gp positive CRC cells by decreasing the overexpression of *P-gp*. Moreover, in other types of cancer cells that are P-gp-positive, namely breast cancer or ovarian cancer cells, this combination also shows synergistic inhibitory effect. Berberine did not enhance the cytotoxicity of evodiamine in normal cells (Guan et al., 2020). In a different study, although evodiamine displayed antiproliferative and antimigratory activities in human gastric cancer (AGS) cells, it promoted metastasis by the concomitant increase in IL-8 secretion and mRNA expression of vascular cell adhesion protein. Further, berberine was shown to counteract this side-effect and maintain the antiproliferative and antimigratory properties of evodiamine to improve its therapeutic effects (Shi et al., 2013).

San-Huang-Xie-Xin Decoction, containing *Rhei Rhizoma*, *C. Rhizoma*, and *Radix Scutellariae* at a ratio of 2:1:1 (w/w/w), was reported to regulate p53 signaling pathway related genes and caused apoptosis and DNA damage in the HCC (HepG2) cells. The main pharmacological effect might be due to the presence of *C. Rhizoma* (Cheng et al., 2008).

Chingwaysan (Qingweisan), a well-known Chinese herbal formula, consists of *Angelicae Sinensis* Radix, *Rehmanniae Radixet Rhizoma*, *Moutan Radicis Cortex*, *C. Rhizoma*, and *Cimicfugae Rhizoma* at a ratio of 3:3:5:5:10 (w/w/w/w/w). Chingwaysan inhibits cell viability by inducing cell apoptosis via the Bax signaling pathway in human oral cancer (OC2 and TSCCa) cells (Liao et al., 2005).

Gegen Qinlian Decoction, containing *Radix Puerariae*, *Radix Scutellariae*, *C. Rhizoma* and *Glycyrrhiza* at a rate of 5:3:3:2 (w/w/w/w), is frequently used to treat UC by means of its anti-inflammatory and antioxidative properties and inhibition of the transcription of oncogenes (Wei M. et al., 2020). It not only exerts antitumor effects when used alone, as described in a recent review (Lu et al., 2021), but also improves clinical symptoms and reduces adverse events in combination with western medicines (Fan Y. et al., 2019).

Xiao-Xian-Xiong Decoction contains *C. Rhizoma*, *Pinellia ternate*, and *Fructus trichosanthis*. It induces proliferative switching by inducing G_0_/G_1_ phase arrest via the inhibition of Facilitates chromatin transcription (FACT) and c-MYC transcription in quiescent lung cancer *in vitro* and *in vivo* (Bi et al., 2020).

Prostacaid is a 33-ingredient dietary supplement with a mixture of vitamins, minerals, multi-botanical extracts, and derivatives, which hinders abnormal cell proliferation and promotes cell apoptosis in androgen-independent or dependent prostate cancer of mouse and human. *C. Rhizoma* is one of the ingredients (Yan and Katz, 2010). Prostacaid suppresses cell proliferation by inducing G_2_/M phase arrest, induction of apoptosis and regulating the expression of CCND1, CDK4, CDKN1A, E2F1, MAPK6, and PCNA genes (Yan and Katz, 2010; Jiang et al., 2011). Furthermore, it also inhibits the metastatic behavior of the human prostate cancer cells by inhibiting cell adhesion, invasion, and invasion, by downregulating of the expression of the *CAV1*, *NR2F1*, *PLAU*, and *IGF2* genes and suppressing the secretion of urokinase plasminogen activator (Jiang et al., 2011).

### Other Combinations

Besides being used in combination with chemotherapeutic drugs and botanical drugs, berberine is also found to have protective effects against radiotherapy. Berberine can simultaneously act as a radiosensitizer and a photothermal agent to supplement effects of chemo-radiotherapy of liver cancer when it was loaded into folic acid with Janus gold mesoporous silica nanocarrier in advance (Li XD. et al., 2019). In addition, berberine can sensitize human HCC cells to ionizing radiation therapy by blocking autophagy at LC3-II manner and inducing G_2_/M phase arrest via the upregulation of mitochondrial oxidative stress and the inhibition of ATP levels, resulting in senescence (Ramesh et al., 2020). Additionally, berberine induces DNA repair by affecting DNA repair protein XRCC1-mediated base excision repair to enhance the sensitization of breast cancer cells to cisplatin, camptothecin, and methyl methanesulfonate (Gao et al., 2019). Other sensitization enhancements were also found in human ovarian cancer cells to cisplatin and the radiosensitivity of esophageal squamous cancer after berberine treatment (Yang X. et al., 2013; Chen et al., 2015).

Taken together, *C. Rhizoma* have potent anticancer effects against gastrointestinal and other cancers, as shown in Table 1.

**TABLE 1:** Anticancer properties of *Coptidis Rhizoma* (*C. Rhizoma*).

Bioactive constituents	Anticancer effects	*Cancer* types	*In vitro* models	*In vivo* models	Underlying mechanisms	References
Water extract of *C. Rhizoma*	Induction of apoptosis	Oral cancer	IHOK, HaCaT, HNSCC4 (= HN4), and HNSCC12 (= HN12) cells; Dose: 1–100 μg/ml		Induce mitochondrial Cytochrome C release and Caspase-3 activation	Lee et al. (2006)
	Inhibition of metastasis	Liver cancer	MHCC97-L cells; Dose: 2–512 µM		Downregulate Rho/ROCK signaling pathway Reduce F-actin polymerization; Damage cytoskeleton network	Wang et al. (2010a)
	Epigenetic regulation		MHCC97-L; Dose: 1.75–448 μg/ml		Upregulate miR21 and miR23a	Zhu et al. (2011)
Water extract of *Coptis japonica* Makino	Inhibition of metastasis		HUVECs; Dose: 1–100 μg/ml	Male Sprague-Dawley rats (7 weeks old), the three-dimensional rat aortic ring sprouting assay *ex vivo*; Dosage: 10 or 25 μg/ml	Inhibit vascular endothelial growth factor (VEGF)-induced MMP-2 and MMP-9 expressions; Inhibit VEGF-induced tube formation *in vitro* and micro-vessel sprouting *ex vivo*	Kim et al. (2016b)
	Cell cycle arrest				Induce G_0_/G_1_ phase arrest; decrease the expression of Cyclin D, Cyclin E, CDK2, and CDK4	
Methanol extract of *C. Rhizoma*	Induction of apoptosis	Gastric cancer	SNU-668; Dose: 100 μg/ml		Activate Bax-dependent Caspase-3	Park et al. (2005)
		Colorectal cancer	SNU-C4; Dose: 10–500 μg/ml		Activate Caspase-3	Kim et al. (2004)
Extracted *C. Rhizoma* powder	Induction of apoptosis	Liver cancer	HepG2 cells; Dose: 0.125–4 mg/ml		Downregulate Bcl-2; Activate Caspase-3, Caspase-9, PARP; Upregulate the expression of Egr-1 and NAG-1 proteins	Auyeung and Ko, (2009)
	Cell cycle arrest				Induce G_2_/M phase arrest	
*C. Rhizoma* granules	Induction of apoptosis	Glioma	U251, U87, H4, LN229, and BV2 cells; Dose: 0.3125–10 mg/ml	Balb/c nude mice (5 weeks old, 18–22g), subcutaneously injected U87 cells; Dosage: 10–20 mg/per mouse (i.g.)	Reduce total Caspase-3 and induce cleavage Caspase-3	Li et al. (2017b)
	Regulation of signal transduction				Inhibit STAT3 phosphorylation	
	Inhibition of metastasis				Lower HDAC3 expression	
	Cell cycle arrest				Induce G_2_/M phase arrest	
*C. Rhizoma* extract	Induction of apoptosis	Malignant melanoma	A2058, UACC257, UACC62, MeWo, SK-Mel-2, M14, and Malme3M cells; Dose: 50–100 μg/ml		Suppress BCL2A1, Mcl-1, and Bcl-w; increase Bax and Bak	Xu et al. (2017)
	Cell cycle arrest	Squamous cell carcinoma	KB and SCC-25 cells; Dose: 20–400 μg/ml	Nude mice, inoculate kB cells; Dosage: 250 mg/kg (p.o.)	Mediate CDK4, CDK6, Cyclin B1, Cyclin E, Cyclin D1, and p27	Wang et al. (2011)
	Inhibition of metastasis				Mediate E-cadherin and osteopontin	
	Regulation of signal transduction				Target STAT3, p53, and BRCA1; Regulate Raf-1, ERK1/2, p38, and ERK; Regulate PI3K signaling pathway by targeting AKT and PTEN.	
Berberine	Induction of autophagy	Gastric cancer	BGC-823 cells; Dose: 14–108 μM	Female BALB/c-nu nude mice (18–22 g), inject BGC-823 cells; Dosage: 5–20 mg/kg (i.p.)	Active cytostatic autophagy by upregulating Beclin-1 and microtubule-associated protein 1 LC3-II, and inhibit mTOR/p70S6K, AKT and MAPK (ERK, JNK and p38) signaling pathway	Zhang et al. (2020a)
		Acute lymphoblastic leukemia	EU-6 and SKW-3 cells; Dose: 0–100 μM	The NOD-SCID mice, inject EU-6 cells to establish the ALL xenograft mice; Dosage: 10 mg/kg/d (p.o.)	Promote autophagic cell death and ameliorates the conditions of disease by inactivating AKT/mTORC1 signaling pathway	Liu et al. (2020a)
		Acute myeloid leukemia	Jurkat and U937 cells; Dose: 100 µM	Male NOD/SCID mice (6–8 weeks old), give radiation at a sublethal dose (1.0 Gy/min) for 1 min and caudal vein inject of Jurkat cells (2 × 10^6^ cells); Dosage: 20 mg/kg (i.g.)	Downregulate MDM2 expression in p53-deficient leukemic cells and induce pro-apoptotic effect in p53-deficient leukemic cells	Liu et al. (2020c)
		Glioblastoma	U87 and U251 cells; Dose: 10 µM	ALB/c nude mice (6–8 weeks old), inject U87/TMZ-R cells; Dosage: 50 mg/kg (i.p.)	Reduce temozolomide resistance by augmenting autophagy via ERK1/2 signaling pathway	Qu et al. (2020)
			U87MG cells; Dose: 10–250 µM		Induce oxidative stress	Palma et al. (2020)
		Liver cancer	Huh-7 cells; Dose: 0–400 µM		Augment cell apoptosis and necrosis by inhibiting autophagy via targeting reactive oxygen species and LC3-II in HCC Huh-7 cells infected with hepatitis C virus RNA.	Tai et al. (2020)
			HepG2 cells; Dose: 50–200 µM MHCC97-L cells; Dose: 100–400 µM		Inhibit AKT and enhance P38 MAPK signaling to inhibit the mTOR-signaling pathway; Suppress Bcl-2 expression to activate Beclin-1 and Bax	Wang et al. (2010b)
		Breast cancer	MCF-7 cells; Dose: 10 μM with 7.2 J/cm2	BALB/c nude female mice (5 weeks old), inject MCF-7/ADR cells; Dosage: 10 mg/kg (i.g.)	Inhibit autophagy by modulating the PTEN/AKT/mTOR signaling pathway to reverse doxorubicin resistance	Wang et al. (2020)
	Immune balance	Liver cancer	Bone marrow cells from tibias and femurs of mice; Dose: 10 μM	C57BL/6J male mice (6–8 weeks old), treated by ethanol; Dosage: 10–100 mg/kg (i.g.)	Activate G-MDSC-like population in mice liver; Alleviate alcohol-induced hepatic damage; Suppress acute-on-chronic damage in mice by regulating the G-MDSC-like population via IL-6/STAT3 signaling pathway; Regulate gut microbial community	Li et al. (2020d)
				Male Wistar rats (8–10 weeks old), treated by doxorubicin; Dosage: 60 mg/kg (i.p.)	Increase TLR2 expression; decrease the expression of TLR4, NF-κB, IL-6, IL-10, IL-12, MCP-1, TNF-a, IFN-g induced by DOX; Reduce cytochrome P450s (CYP) expression	Sun et al. (2020a)
		Diffuse large B-cell lymphoma	LY1, LY3, LY8, Val, and U2932 cells; Dose: 0–60 μM	Male BALB/c mice (6 weeks old, 18 g–20 g), inject A20 cells; Dosage: 100 mg/kg (i.g.)	Modulate c-myc/CD47 axis; Downregulate CD47 expression at transcriptional level by suppressing c-myc expression; Enhance the phagocytosis of macrophages to eliminate tumor cells; Enhance the efficiency of anti-CD47 antibody and rituximab-mediated phagocytosis	Ren et al. (2021)
		Non-small cell lung cancer	A549, H157, H358, H460, H1299, and H1975 cells; Dose: 5 and 10 μM	Female C57BL/6 mice (8 weeks old), inoculate Lewis cells; Dosage: 4 and 8 mg/kg (i.p.)	Reduce PD-L1 expression; Promote antitumor immunity by inhibiting the deubiquitination activity of COP9 signalosome 5	Liu et al. (2020g)
	Anti-inflammation		Raw 264.7 cells; Dose: 3.345 mg/ml in the methanol-ethanol solution		Attenuate inflammation in the early phase; Interact with TLR4; Interfere with TLR4/MyD88/NFκB signaling pathway	Gong et al. (2019a)
			THP-1 cells; Dose: 10 μM	A *NEK7* knockdown mouse model; Dosage: 5 mg/kg (i.p.)	Directly target the NEK7 protein; Block NEK7−NLRP3 interaction; Prevent IL-1β release	Zeng et al. (2021)
			Autoreactive inflammatory CD4^+^ T cells (T helper (Th)1 and Th17 subtypes)		Directly inhibit the functions of pro-inflammatory Th1 and Th17 cells and their differentiation; Indirectly reduce Th cell-mediated inflammation by regulating or inhibiting other cells; Contribute to autoreactive inflammation such as Tregs	Ehteshamfar et al. (2020)
		Colorectal cancer	Intestinal surface epithelial cells (IECs)-18 rat intestinal epithelial cells were treated with LPS; Dose:100 µM		Mediate TLR4/NF-κB and MAPK/AP-1 pathway; Regulate Bax/Bcl-2 gene expression; Downregulate cathepsin and IAPs; Cause mitochondria to release excessive levels of Cytochrome C	Xu et al. (2020)
			FHC cells; Dose: 5 µM	Acute colitis: 6-week old male mice, treated by DSS Colitis-associated colon cancers: six-week-old male C57BL/6 mice, treated by AOM and DSS; Dosage: 28 mg/kg (p.o.)	Recover Dicer expression	Wu et al. (2020)
			Caco-2 cells	Male Sprague-Dawley rats (∼250 g), treated by DSS; Dosage: 40 mg/kg (i.g.)	Regulate the levels of intestinal microbiota-associated tryptophan metabolites; Activate aryl hydrocarbon receptor; Improve the disrupted gut barrier function	Jing et al. (2021)
				Female Balb/c mice (7–9 weeks old), treated by DSS; Dosage: 50 mg/kg (p.o.)	Activate mTORC1 pathway; Elevate the proportion of Treg cells	Li et al. (2019a)
			HT-29; Dose: 0.1–30.0 µM		Inhibit COX-2 both at the mRNA and protein levels; Reduce COX-2 activity and prostaglandin E2 concentration	Tai and Luo, (2003)
			SW620 and LoVo cells; Dose: 5–80 µM	Male BALB/C nude mice (4–6 weeks old, 18 ± 2g), inject CRC cell; Dosage: 50–200 mg/kg (i.g.)	Attenuate COX-2/PGE2 expression; Inhibit the phosphorylation of JAK2 and STAT3; decrease MMP-2 and MMP-9 expression	Liu et al. (2015a)
			HCT116 cells, Raw 264.7 macrophage; Dose: 25 μM	C57BL/6J-ApcMin/+ mice (4 and 8 weeks old), treated by DSS, Dosage (4 weeks old-mice): 1 mg/ml (p.o.), Dosage (8 weeks old-mice): 50 mg/kg (i.g.)	Participate in inflammatory response-driven EGFR signaling pathway	Li et al. (2017a)
			RAW 264.7 macrophage; Dose: 10 µM	C57BL/6, treated by DSS; Dosage: 20 mg/kg (i.g.)	Bind to cytosolic phospholipase A2a (PLA2G4A) directly; Inhibit PLA2G4A activity; Suppress MAPK/JNK signaling pathway; Ameliorate colonic inflammation	Zhai et al. (2020)
			Cardiac blood mononuclear cells; Dose: 1–10 µM	Male Sprague-Dawley rats (180–220 g), treated by TNBS; Dosage: 7.5 or 15 mg/kg (p.o.)	Exert a protective effect on UC by regulating the interaction between enteric glial cells and intestinal epithelial cells-immune cells; Inhibit IL-8 production in rectal mucosa	Zhou and Mineshita, (2000)
			CCD-18Co, U937, THP-1, T lymphocyte, Jurkat T cells. Dose: 50 μM	Wild-type male C57BL/6 mice (8 weeks old, 22–24 g), treated by DSS; Dosage: 50 mg/kg (p.o.)	Interfere with mucosal inflammation driven by oncostatin M (OSM); Attenuate intestinal inflammation; Protect intestinal barrier function; Restore tissue remodeling and fibrosis; decrease inflammatory infiltrations; Mediate JAK-STAT, MAPK, and AKT signaling pathways	Li et al. (2020c)
				Male wistar rats (200–230 g), treated by DSS; Dosage: 10–50 mg/kg (i.g.)	Inhibit IL-6/STAT3/NF-κB signaling pathway	Zhu et al. (2019a)
		Human non-small cell lung cancer	A549; Dose: 0–50 μM		Induce quiescence and apoptosis by modulating cell cyclins (A1, A2, B, D1) and inflammation	Kumar et al. (2020)
		Breast cancer	MDA-MB-231; Dose: 5–50 µM		Inhibit the phosphorylation of c-Jun and c-Fos; Reduce the expressions of TNF-α and IL-6; Suppress the activation of NF-κB; Prevent IκBα from degradation	Zhao and Zhang, (2020)
	Gut microbiota balance	Colorectal cancer		Female C57BL/6 mice (18–20 g), treated by AOM/DSS; Dosage: 100 mg/kg (i.g.)	Alter metabolic and the composition of gut microbiota at the phylum and genus levels	Chen et al. (2020c)
				Male Sprague-Dawley rats (60–180 g), treated by DSS; Dosage: 100 mg/kg (i.g.)	Elevate lactic acid-producing bacteria and carbohydrate hydrolysis bacteria; Reduce conditional pathogenic bacteria to treat colonic damage	Liao et al. (2020)
			Caco-2 cells	Male Sprague-Dawley rats (∼250 g), treated by DSS; Dosage: 40 mg/kg (i.g.)	Alleviate DSS-induced colitis; Activate AhR; Adjust tryptophan metabolite levels associated with the gut microbiota	Jing et al. (2021)
				Female Balb/c mice (18–22 g, 30–40 days old), treated by DSS; Dosage: 40 mg/kg (p.o.)	Mediate the balance of Treg/Th17 cells; Regulate the intestinal flora in the colon	Cui et al. (2018)
			Intestinal facultative anaerobes of Male Sprague–Dawley (SD) rats (180–200 g); Dose: 10–20 μg/ml	Male hamsters (140–160 g), Dosage: 100 mg/kg/d (p.o.) The ob/ob mice (40–50 g); Dosage: 100 mg/kg/d (p.o.), 20 mg/kg (i.p.)	Increase the abundance of butyrate-producing bacteria; Indirectly change the composition of intestinal bacterial mice. Indirectly increase butyrate; Inhibit NADH and bacterial ATP production, increase levels of phosphotransbutyrylase/butyrate kinase and butyryl-CoA (acetate-CoA transferase)	Wang et al. (2017)
				Male sprague–Dawley rats (5 weeks old); Dosage: 100 mg/kg (i.g.)	Increase butyrate and glutamine levels in feces; Enrich the abundance of *Firmicutes*; decrease *Proteobacteria* at the phylum level; increase the proportion of *unclassified_f_* Porphyromonadaceae*, unclassified_f_* Lachnospiraceae*, Lactobacillus* and *unclassified_o_ Clostridiales*, at the genera level	Chen et al. (2020a)
	MicroRNA	Multiple myeloma	U266 multiple myeloma cells; Dose: 40–160 µM		Increase Set9; Suppress NF-κB, miR-21 and Bcl-2 levels; Stimulate ROS generation	Hu et al. (2013)
			RPMI-8266 cells; Dose: 75 μM. U266 cells; Dose: 120 μM		Decrease IL-6 and STAT3; Suppress miR-21 level; Upregulate PDCD4	Luo et al. (2014)
			Human multiple myeloma cell RPMI-8266 and U266		Downregulate miR-106b/25 (*in silico*)	Gu et al. (2017)
		Liver cancer	HepG2 cells; Dose: 40 µM		Upregulate miR-21-3p; Modulate the expression of methionine adenosyltransferase 2A and methionine adenosyltransferase 2B	Lo et al. (2013)
			HepG2 cells; Dose: 0–300 μM		Increase miR-22-3p; Target Sp1	Chen et al. (2016b)
			HepG2 cells (p53 wild type), Hep3B cells (p53-deficient); Dose: 100 μM	Female BALB/c nu/nu athymic nude mouse (6 weeks old), subcutaneous inject of MHCC97L cells; Dosage: 10 mg/kg (i.p.)	Induce miR-23a expression; Suppresses Nek6 (NIMA Related kinase 6)	Wang et al. (2014b)
		Colorectal cancer	HCT116 cells; Dose: 1–100 µM		Inhibit miR-21 expression; Mediate miR-21-integrin β4-PDCD4 pathway	Lü et al. (2018)
		Gastric cancer	SGC-7901, BGC-823, cisplatin-resistant mutants SGC-7901/DDP, and BGC-823/DDP cells; Dose: 10 μM		Upregulate miR-203; Target Bcl-w; Activate caspases	You et al. (2016)
			SGC-7901 cells; Dose: 2.5–30 µM		Regulate Ras and Jak-STAT signaling pathways	Yang et al. (2018)
		Esophageal squamous cell carcinoma (ESCC)	KYSE-450, TE-1, and Eca109 cells; Dose: 5–10 μM		Downregulate miR-212	Chen et al. (2019)
		Ovarian cancer	SKOV3 and OVCAR3 cells; Dose: 10 μM		Increase cell sensitivity to cisplatin via miR-21/PDCD4 axis; decrease the expression and function of miR-21; Enhance PDCD4 level	Liu et al. (2013b)
	Induction of apoptosis	Liver cancer	HepG2 cells; Dose: 10–300 µM		Mediate AKT-ASK1-ROS-p38MAPKs-linked cascade	Hyun et al. (2010)
			HepG2 cells; Dose: 10–100 µM		Activate nonsteroidal anti-inflammatory drug (NSAID)-activated gene-1 (NAG-1)	Auyeung and Ko, (2009)
			MHCC97-H and HepG2 cells; Dose: 50–200 µM		Inhibit AKT and PI3K levels	Song et al. (2019)
			WRL68 and Huh7 cells; Dose: 5–30 µM		Upregulate Bax, Bid, CIDEA, HRK, and p21; Downregulate AKT and Bcl-2; Inhibit survivin gene expression; Activate Caspase-9, Caspase-3, and Caspase-7	Yip and Ho, (2013)
			HepG2 cells; Dose: 50–200 µM MHCC97-L cells; Dose: 100–400 µM		Augment Bax expression and Cytochrome C release; Activate Caspases-3 and Caspase-9	Wang et al. (2010b)
		Colorectal cancer	SW620 cells; Dose: 5–50 µM		Induce ROS generation; Activate JNK/p38 MAPK and FasL pathways	Hsu et al. (2007)
			IMCE cells; Dose: 50–200 µM		Mediate the MEK/ERK and B-Raf signaling pathways; Activate apoptosis-inducing factor (AIF) to product ROS; Induce two targets of ROS production (cathepsin B release from lysosomes and PARP activation)	Wang et al. (2012)
		Gastric cancer	BGC-823 and SGC7901 cells; Dose: 10–100 µM	Female BALB/C nu/nu nude mice (6 weeks old), inject BGC-823 cells; Dosage: 10 mg/kg (inject intratumorally)	Suppress Akt/mTOR/p70S6/S6 pathways	Yi et al. (2015)
		Cholangiocarcinoma	QBC939 cells; Dose: 10–80 µM		Increase Bax expression; decrease Bcl-2 and Bcl-xL expression	He et al. (2012b)
		Pancreatic cancer	PANC-1 and MIA-PaCa2 cells; Dose: 1–15 µM		Induce ROS generation	Park et al. (2015)
		Glioblastoma	U87MG cells; Dose: 10–250 µM		Induce oxidative stress	Palma et al. (2020)
		Osteosarcoma	MG-63 cells; Dose: 20–80 µM		Induce accumulation of DNA double-strand breaks	Zhu et al. (2014b)
		Prostate cancer	LNCaP, DU145, PC-3, and PWR-1E cells; Dose: 10–100 µM		Activate Caspase-3	Mantena et al. (2006)
			PC-3, LNCaP, and PWR-1E cells; Dose: 25–75 µM		Induce ROS generation	Meeran et al. (2008)
		Non-small cell lung cancer	A549 cells; Dose: 30–200 µM		Suppress MMP-2 and Bcl-2/Bcl-2-associated X protein (Bax) signaling pathways	Li et al. (2018)
	Regulation of signal transduction	Colorectal cancer	HCT116 and SW480 cells; Dose: 25–800 μM	Male BALB/c nude mice (4 weeks old), inject HCT116 cells; Dosage: 30–120 mg/kg (i.g.)	Inhibit PI3K/AKT signaling pathway; Downregulate insulin-like growth factor 2 (IGF2) mRNA-binding protein 3 (IGF2BP3)	Zhang et al. (2020c)
			SW620 and LoVo cells; Dose: 5–80 μM	Male BALB/C nude mice (4–6 months old, 18 ± 2g), subcutaneously inject CRC cells; Dosage: 50–200 mg/kg (i.g.)	Mediate COX-2/PGE2 and JAK2/STAT3 signaling pathways; Downregulate MMP-2 and MMP-9 expression	Liu et al. (2015a)
		Liver cancer	MHCC97-H and HepG2 cells; Dose: 50–200 µM		Inhibit phosphorylation of AKT and PI3K	Song et al. (2019)
		Several tumor types including breast, prostate, colon, ovarian, melanoma, endometrial, pancreatic, and lung cancers	SK-BR-3, MCF-7, and T47D cells; Dose: 0–2 µM		Inhibit the Erythropoietin-producing hepatocyte B4 (EphB4)	Zhu et al. (2019b)
	Cell cycle arrest	Colorectal cancer	HCT116 and SW480 cells; Dose: 25–800 μM		Induce G_0_/G_1_ phase arrest; Downregulate IGF2BP3; Repress PI3K/AKT pathway	Zhang et al. (2020c)
		Breast cancer	SKBR-3, BT-474, T47D, MDA-MB-231, and MCF-7 cells; Dose: 10–100 μM		Induce G_0_/G_1_ phase arrest; Downregulate cyclins A, D1, and E	Kuo et al. (2011)
		Cholangiocarcinoma	QBC939 cells; Dose: 10–80 µM		Induce G_0_/G_1_ phase arrest; increase the expression of Cip1/p21 and Kip1/p27; decrease the expression of Cdk2, Cdk4 and cyclins D1, and the activity of the Cyclins-Cdk complex	He et al. (2012b)
		Glioblastoma	U87MG cells; Dose: 10–250 µM		Induce G_0_/G_1_ phase arrest	Palma et al. (2020)
		Prostate carcinoma	LNCaP, DU145, PC-3, and PWR-1E cells; Dose: 10–100 µM		Induce G_0_/G_1_ phase arrest; Inhibit the expression of cyclins (D1, D2, E), Cdk (2, 4, 6) proteins; increase the expression of the Cip1/p21 and Kip1/p27; Enhance binding of Cdk inhibitors to Cdk	Mantena et al. (2006)
	Inhibition of metastasis	Breast cancer	MCF-7, MDA-MB-231 cells; Dose: 1–200 μM		Regulate Metadherin (MTDH) expression	Sun et al. (2019b)
			ZR-75–30, HEK293, and SMCC-7721 cells; Dose: 0–50 μM		Downregulate the expression of Ephrin-B2, Syntenin 1, PICK1, MMP-2, and MMP-9; Inhibite the phosphorylation of VEGFR2 and AKT.	Ma et al. (2017)
		Ovarian cancer	SKOV3 and 3AO cells; Dose: 2.5–320 µM		Increase miR-145 expression; decrease MMP16 expression	Li et al. (2021e)
		Colorectal cancer	SW620 and LoVo cells; Dose: 10–80 µM	Male BALB/C nude mice (4–6 weeks old, 18 ± 2g), inject CRC cells; Dosage: 50–200 mg/kg (i.g.)	Mediate COX-2/PGE2 and JAK2/STAT3 signaling pathways	Liu et al. (2015a)
	Epigenetic regulation	Multiple myeloma	U266 cells; Dose:40–120 µM		Inhibit the expression of DNMT1 and DNMT3B; Alter the CpG methylation of *p53*	Qing et al. (2014)
		Colorectal cancer	Colon tissue from neonatal rats; Dose: 15 µM		Increase the expression of DNMT1, DNMT3A, DNMT3B and miR-152, miR-429, miR-29a	Huang et al. (2017a)
		Lung cancer	A549 cells; Dose: 20–200 µM		Downregulate histone deacetylases; decrease the expression of mRNA and protein of MMP-2 and MMP-9	Kalaiarasi et al. (2016)
Berberine-mediated photodynamic therapy (PDT)	Induction of autophagy	Malignant melanoma	A375 cells and SK-MEL-19 cells; Dose: 10 μM with 7.2J/cm^2^		Induce Caspase-3 activation and ROS release; increase LC3-related autophagy; Activate endoplasmic reticulum stress	Fang et al. (2021)
Berberine coupled with exercise	Induction of apoptosis	Breast cancer	MCF7 cells; Dose: 50–150 μg/ml	BALB/c mice, establish the model of orthotopic transplantation for 4T1 breast cancer; Dosage: 45–145 mg/kg (i.g)	Activate Fas death receptor pathway	Ma et al. (2020)
Hange-shashin-to (HST) (include berberine)	Anti-inflammation	Colorectal cancer		Male Wistar/ST rats (7 weeks old), treated by TNBS; Dosage: 3.75 or 6.5 mg/kg (berberine), 467 or 934 mg/kg (HST) (p.o.)	Inhibit lipopolysaccharide-induced cytokine production; Activate MAPK and NF-κB in macrophages	Kawashima et al. (2004)
Berberine and phenylboronic esters-modified carboxylmethyl chitosan	Anti-inflammation	Colorectal cancer	Use dialysis membrane to evaluate the release of OC-B-BBR micelles under sink conditions; OC-B-BBR micelles Dose: 2 mg	C57BL/6 J mice (6–8 weeks old), treated by DSS; nano-berberine; Dosage: 30 mg/kg (i.g.)	Improve the symptoms of colitis and colon damage; Regulate IL-6 expression; Remodel the intestinal microbiota	Zhao et al. (2021b)
Berberine and Hsp90 inhibitors	MicroRNA	Colorectal cancer	HCT-15 and HT-29 cells; Dose: 10 μM		Suppress the overexpression of CDK4 and miRNA-296-5p; Activate Pin1-β-catenin-cyclin D1 signaling pathway	Su et al. (2015)
Coptisine	Anti-proliferation	Pancreatic cancer	YPK-1, Panc-1 and MiaPaCa-2 cells; Dose: 0.01–100 μg/ml		23 genes (e.g., RP2, PAK1, MMP14) are positive correlated with the ID50 values of coptisine; 4 genes (e.g., SDHC, WBP4, TAGLN2) are inverse correlated with the ID50 values of coptisine. (*in silico*)	Hara et al. (2005)
		Liver cancer	HepG2, Hep3B, SK-Hep1, and PLC/PRF/5 cells; Dose: 1–20 μg/ml			
		Leukaemia	K562, U937, P3H1, and Raji cells; Dose: 1–20 μg/ml			
	Cell cycles arrest	Colorectal cancer	HCT-116 cells; Dose: 0–50 μg/ml	Male HCT-116 xenograft BALB/c nude mouse mode; Dosage: 150 mg/kg (i.g.)	Induce G_0_/G_1_ phase arrest; decrease the expression of CyclinD1, Cyclin E, CDK 4, CDK 2, and the mRNA level of CyclinD1 and Cyclin E proteins	Huang et al. (2017b)
		Osteosarcoma	MG63 cells; Dose: 0–40 µM	Female BALB/C nude mice (18–22 g, 6 weeks old), inject MG63 cells; Dosage: 50 mg/kg (i.p.)	Induce G_0_/G_1_ phase arrest; Downregulate the expression of CDK4 and cyclin D1	Yu et al. (2014)
		Pancreatic carcinoma	PANC-1 cells; Dose: 25–150 µM		Induce G_0_/G_1_ phase arrest and S phase reduction; Inhibit ERK phosphorylation; decrease total ERK levels	Zhang et al. (2020b)
	Induction of apoptosis	Colorectal cancer	HCT-116 cells; Dose: 0–50 μg/ml	Male HCT-116 xenograft BALB/c nude mouse mode; Dosage: 150 mg/kg (i.g.)	Induce caspase-dependent apoptosis through PI3K/Akt and mitochondrial-associated apoptotic pathway	Huang et al. (2017b)
		Liver cancer	SMMC7721, HepG2, BEL7402, HL7702, and H9 cells; Dose: 12.5–100 µM	Male nude mice (5 weeks old); Dosage: 50 mg/kg (i.p.)	Activate 67-kDa Laminin Receptor/cGMP Signaling	Zhou et al. (2018)
	Inhibition of metastasis	Breast cancer	MDA-MB-231 cells; Dose: 16–64 µM		Downregulate MMP-9; increase metalloproteinase 1 (TIMP-1)	Li et al. (2014b)
		Colorectal cancer	HCT-116 cells; Dose: 0–50 μg/ml	Male HCT-116 xenograft BALB/c nude mouse mode; Dosage: 150 mg/kg (i.g.)	Inhibit RAS-ERK pathway	Huang et al. (2017b)
		Osteosarcoma	MG63 cells; Dose: 0–40 µM	Female BALB/C nude mice (18–22 g, 6 weeks old), inject MG63 cells; Dosage: 50 mg/kg (i.p.)	Decrease the expression of VE-cadherin and integrin β3; Diminish STAT3 phosphorylation	Yu et al. (2014)
	Regulation of signal transduction	Colorectal cancer	HCT-116 cells; Dose: 0–50 μg/ml	Male HCT-116 xenograft BALB/c nude mouse mode; Dosage: 150 mg/kg (i.g.)	Inhibit the RAS-ERK pathway	Huang et al. (2017b)
		Osteosarcoma	MG63 cells; Dose: 0–40 µM	Female BALB/C nude mice (18–22 g, 6 weeks old), inject MG63 cells; Dosage: 50 mg/kg (i.p.)	Decrease the expression of VE-cadherin and integrin β3; Diminish STAT3 phosphorylation	Yu et al. (2014)
		Hepatocellular carcinoma	Hep3B cells; Dose: 0–50 µM		Downregulate PI3K/Akt/mTOR signaling pathway; Regulate ROS-mediated mitochondrial dysfunction	Kim et al. (2021)
	Induction of autophagy	Hepatocellular carcinoma	Hep3B cells; Dose: 0–50 µM		Increase LC3-I/II and Beclin-1	Kim et al. (2021)
Palmatine	Anti-helicobacter *pylori* activity	Gastric cancer		Male Sprague-Dawley rats (180–200 g), treated by HCl/ethanol solution; Dosage: 100 mg/kg		Jung et al. (2014)
Epiberberine	Regulation of signal transduction	Gastric cancer	MKN-45 and HGC-27 cells; Dose: 0–80 µM	Male BALB/c nude mice (4 weeks old), inject MKN-45 cells; Dosage: 30–120 mg/kg (p.o.)	Mediate the p53/Bax pathway; decease the expression of Bcl-2, XIAP; increase the expression of p53, Bax, p21, p27; Activate Cytochrome C and Caspase-3	Yu et al. (2020)
		Acute myeloid leukemia	THP-1 and HL-60 cells; Dose: 1–8 μM	Female NOD/SCID mice (18–21 g, 5 weeks old), inject THP-1 cells; Dosage: 10–20 mg/kg (i.v.)	Inhibit lysine specific demethylase 1 (LSD1)	Li et al. (2020e)
	Induction of apoptosis	Gastric cancer			Downregulate the expression of Bcl-2 and XIAP; Upregulate expression level of Bax, p53; Activate Cytochrome C and Caspase-3	Liu et al. (2020d)
	Cell cycles arrest	Gastric cancer	MKN-45 and HGC-27 cells; Dose: 0–80 µM	Male BALB/c nude mice (4 weeks old), inject MKN-45 cells; Dosage: 30–120 mg/kg (p.o.)	Induce S phase arrest (MKN-45 cells); Induce G_0_/G_1_ phase arrest (HGC-27 cells); Mediate p53-dependent mitochondria-associated pathway	Yu et al. (2020)
Oxyepiberberine	Induction of apoptosis	Colorectal cancer	LS-1034 cells; Dose: 2–8 µM	Nude mice, inject LS-1034 cells; Dosage: 80 mg/kg	Inhibit tubulin polymerization	Ning et al. (2021)
	Inhibition of metastasis	Lung cancer	A549 cells; Dose: 2.5–50 µM H1975 cells; Dose: 0–25 µM	Female BALB/c mice (6–8 weeks old), inject 4 T1 cells; Dosage: 0.5–1 mg/kg (i.p.)	Impede TGF-β1-induced EMT; Interfere with Smad3 promoter	Liu et al. (2020f)
Oxyberberine	Anti-inflammation	Colorectal cancer		Male Balb/C mice (22–24 g), treated by DSS; Dosage: 12.5–50 mg/kg	Downregulate the expression of TLR4 and MyD88 proteins; Inhibit IκBα phosphorylation and NF-κB p65 translocation from cytoplasm to nucleus	Li et al. (2020a)
	Gut microbiota balance				Restore the dysbacteria to normal level	
	Regulation of signal transduction				Inhibit TLR4-MyD88-NF-κB signaling pathway	
Berberrubine	Epigenetic regulation	Colorectal cancer	AMC5 and berberrubine-resistant AMC5/B1 cells; Dose: 25–200 µM		Induce DNA cleavage; Downregulate topoisomerase IIα	Kang and Chung, (2002)
Worenine	Regulation of signal transduction	Colorectal cancer	HCT116 and SW620 cells; Dose: 0–80 μM		Balance the Warburg effect via HIF-1α signaling	Ji et al. (2021a)
	Cell cycles arrest				Induce G_2_/M phase arrest	
Dihydroberberine	Anti-inflammation	Colorectal cancer		Male BALB/C mice (6–8 weeks old, 24–26 g), 3% DSS for 8 days to establish acute colitis model by gavage; Dosage: 50 mg/kg	Decrease colonic pro-inflammatory cytokines and immunoglobulin	Li et al. (2021b)
	Regulation of signal transduction				Block the TLR4/MyD88/NF-κB signaling pathway	
Columbamine	Anti-proliferation	Metastatic osteosarcoma	U2OS cells; Dose: 0–40 μM	BALB/C nude mice (6–8 weeks old), inject U2OS cells; Dosage: 60 μg (inject)	Diminish STAT3 phosphorylation	Bao et al. (2012)
	Cell cycles arrest				Induce G_2_/M phase arrest; Downregulate CDK6 gene expression	
	Inhibition of metastasis				Downregulate MMP2 expression	
Jatrorrhizine	Cell cycles arrest	Malignant melanoma	C8161 cells; Dose: 0–320 μM	BALB/C nude mice (6–8 weeks old), inject green fluorescent protein (GFP) -positive C8161 cells; Dosage: 50 μg (s.c.)	Induce G_0_/G_1_ phase arrest; Enhance the expression of p21 and p27 genes	Liu et al. (2013a)
	Inhibition of metastasis				Hinder the expression of VE-cadherin	
Magnoflorine	Cell cycles arrest	Gastric cancer	MGC803, BGC823 and SGC7901 cells; Dose: 0–160 μM Normal gastric epithelium GES1 cells; Dose: 0–320 μM	Male BALB/c nude mice (5–6 weeks old), inject SGC7901 cells; Dosage: 10 mg/kg (i.p.)	Induce G_2_/M phase arrest; decrease the expresson of Cyclin A and Cyclin B1 proteins; increase the expression of p21 and p27 proteins	Sun et al. (2020b)
	Induction of autophagy				Increase autophagosome formation; Upregulate LC3B-II expression	
	Regulation of signal transduction				Induce ROS-related suppression of serine/threonine-AKT signaling	
	Induction of apoptosis				Downregulate the ratio of Bcl-2/Bax; Activate Caspase-3 and PARP.	
Limonin	Induction of Apoptosis	Colorectal cancer	SW480 cells; Dose: 6.25–100 μM		Downregulate the ratio of Bcl-2/Bax; Deplete mitochondrial membrane potential; Alter intracellular calcium content; Induce Cytochrome C release to cytosol; Activate Caspase-3	(Chidambara Murthy et al., 2011) (Fan et al., 2019a)
	Regulation of signal transduction	Liver cancer	HepG2 cells; Dose: 20–100 μM		Mediate the Wnt signaling pathway; Downregulate p53, cyclin D1, and Bcl2; Upregulate the expression of Bax, Caspase-3 and Caspase-9	Langeswaran et al. (2013)
	MicroRNA	Breast cancer	MCF-7 and MDA-MB-231 cells; Dose: 5–20 μM		Upregulate miR-216a-3p expression; Inhibit Wnt/β-catenin pathway	Su et al. (2019)
	Reversal MDR	Leukaemia	Caco-2 cells, human leukaemia (wild-type CCRF-CEM, multidrug-resistant CEM/ADR5000) cells; Dose: 0.32–32 µM		Inhibit P-gp activity	El-Readi et al. (2010)
Wogonin	Cell cycles arrest	Liver cancer	MHCC97L and HepG2 cells; Dose: 0–800 µM	Male BALB/C nude mice (5 weeks old), inject MHCC97L-luciferase cells; Dosage: 25–50 mg/kg (i.p.)	Promote Cyclin D1 degradation; Activate the glycogen synthase kinase-3β	Hong et al. (2020)
Pinoresinol	Regulation of signal transduction	Colorectal cancer			Moderate PI3K/Akt/mTOR axis (*in silico*)	Sain et al. (2021)
Secoisolariciresionol	Induction of autophagy	Colorectal cancer	SW480 cells; Dose: 40–200 μM		Activate Caspase-3-mediated apoptosis	Ozgocmen et al. (2021)
Vanillic acid	Regulation of signal transduction	Colorectal cancer	HCT116 Cells; Dose: 0–30 µM	Male Balb/c nude mice (4–5 weeks old, 20 ± 2 g), inject HCT116 cells; Dosage: 10–30 mg/kg (p.o)	Suppress HIF-1α expression; Inhibition the mTOR/p70S6K/4E-BP1 and Raf/MEK/ERK Pathways	Gong et al. (2019b)
	Cell cycles arrest				Induce G_0_/G_1_ phase arrest	
	Inhibition of angiogenesis				Inhibit the expression of VEGF and erythropoietin (EPO) proteins; Disrupt tube formation	
Demethylenetetrahydroberberine	Anti-inflammation	Liver cancer	HL7702 cells; Dose: 10–15 µM	Adult male C57BL/6 mice (8 weeks old), induced by a methionine- and choline- deficient (MCD) diet to establish NAFLD animal model; Dosage: 50–150 mg/kg (i.g.)	Repress the NOD-like receptor protein 3 (NLRP3) inflammasome and oxidative stress; Downregulate the expression of TNF-α, IL-1β, IL-6, TGF-β1, α-SMA, collagen 1A1, CYP2E-1, and ATF-4; Mediate the TLR4/NF-κB signaling; Repress the overexpression of ROS and endoplasmic reticulum stress	Zhang et al. (2021b)
	Regulation of signal transduction					
	Reduction of oxidative stress and ER stress					

## Clinical Research

Clinical research on *C. Rhizoma* has mostly focused on berberine in colorectal adenomas and NSCLC. Since 2002, over 2000 participants have taken part in the clinical trials for cancer therapy with berberine. Published results show that berberine exerts protective effects on lung damage caused by radiation *via* intercellular adhesion molecular-1 (ICAM-1) and TGF-β1 in patients with lung cancer. In a randomized, double-blind study, 90 sampled patients with NSCLC were divided into two groups. Berberine significantly decreased the incidence of radiation-induced lung damage as well as the levels of soluble ICAM-1 and TGF-β1, and improved pulmonary function (Liu et al., 2008a). Radiation-induced acute intestinal symptoms (RIAISs) are the most relevant complication that happened in patients of abdominal or pelvic radiotherapy. Berberine exerts preventive effects on acute RIAIS. In one study, 42 randomly sampled patients with cervical cancer were divided into two groups. Patients who had more severe symptoms were in the control group. The study illustrated that berberine could reduce the incidence and severity of RIAISs (Li et al., 2010).

Collectively, there are few clinical studies evaluating *C. Rhizoma* and its active ingredients, most of which are concentrated on berberine, as shown in Table 2. In clinical trials, berberine is generally used as an anticancer adjuvant in NSCLC rather than a major chemotherapy agent as in colorectal adenomas. We look forward to many more studies evaluating the application of *C. Rhizoma* in gastrointestinal cancer in the future.

**TABLE 2:** Clinical trials of berberine against cancers.

Compound	*Cancer* type	Status	Phase	Treatment	Participants	Control	Combination	References
Berberine hydrochloride	Colorectal Adenomas	2017-present	II, III	300 mg tablet by mouth, 2 times/day for 3 years	1,000	Placebo	—	ClinicalTrials.gov Chen et al. (2020c)
		2014–2018 Completed		300 mg, 2 times/day for 2–3 years	1,108		—	
		2017-present		100 or 300 mg tablet by mouth, 2 times/day for 6 months	100		—	
Berberine Sulfate	Advanced Non-small Cell Lung *Cancer* (EGFR Mutation)	2018-present	II	50 mg, p.o., 3 times/day (tid)	50	—	Gefitinib (250 mg p.o., daily)
Berberine	Non-small Cell Lung *Cancer*	2004–2006 Completed	—	20 mg/kg, once a day for 6 weeks	90	Placebo	Radiation therapy (Once a day, 2-Gy to total 60–70 Gy)	Liu et al. (2008b)
	Seminoma	2002–2008 Completed		300 mg tablet, p.o., 3 times/day for 5 weeks	7		Radiation therapy (1.8-Gy/fraction to total 36 Gy)	Li et al. (2010)
	Lymphomas				29			
	Cervical *Cancer*				42		Radiation therapy (2-Gy/fraction to total 46 Gy)	

Note: per os (p.o.), third in die (tid), Gray (Gy), and epidermal growth factor receptor (EGFR).

## Conclusion and Future Perspective

*Cancer* is a complex systemic chronic disease threatening human health and quality of life. As a traditional Chinese medicine for the treatment of gastrointestinal disorders, *C. Rhizoma* has the unparalleled advantage in treating digestive system cancers. The percentage of estimated deaths of digestive system cancers is increased in recent years (25.69%, 153,030/595,690 for year 2016; 26.24%, 157,700/600,920 for year 2017; 26.38%, 160,820/609,640 for year 2018; 27.26%, 165,460/606,880 for year 2019; 27.66%, 167,790/606,520 for year 2020; 27.82%, 169,280/608,570 for year 2021) according to the cancer statistics over the past 5 years in the United States. The anticancer research advance of *C. Rhizoma* highlights the need for a systematic review.

From the current advances of using *C. Rhizoma* for the treatment of gastrointestinal and other cancers, some achievements and limitations could be appreciated, as well as the most promising direction for future research. Most of the studies are mainly *in vitro*, and there are a few *in vivo* investigations, especially in human clinical trials. In the limited clinical studies on *C. Rhizoma* and its active ingredients, berberine has been used as an anticancer adjuvant. In fact, several types of secondary metabolites of *C. Rhizoma* are in need of in-depth study for their properties, that would be clinically valuable and might represent a research hotspot in the future. The chemical structures of the active ingredients from *C. Rhizoma* have been investigated to improve their pharmacological activities, which commonly results in modification of the molecular structure and OMICS approaches in natural product research. In order to obtain superior physical and/or chemical properties and bioavailability, novel drug delivery systems have been a focus of active research and shown great promise, such as hybrid microparticle of berberine, synthesized silver nanoparticles combined with aqueous extract of *C. Rhizoma*, berberine nanoemulsion and more. Moreover, combinations with other drugs or therapies aiming at enhancing efficacy and reducing toxicity is also a significant way to improve clinical outcomes.

**FIGURE 2:**
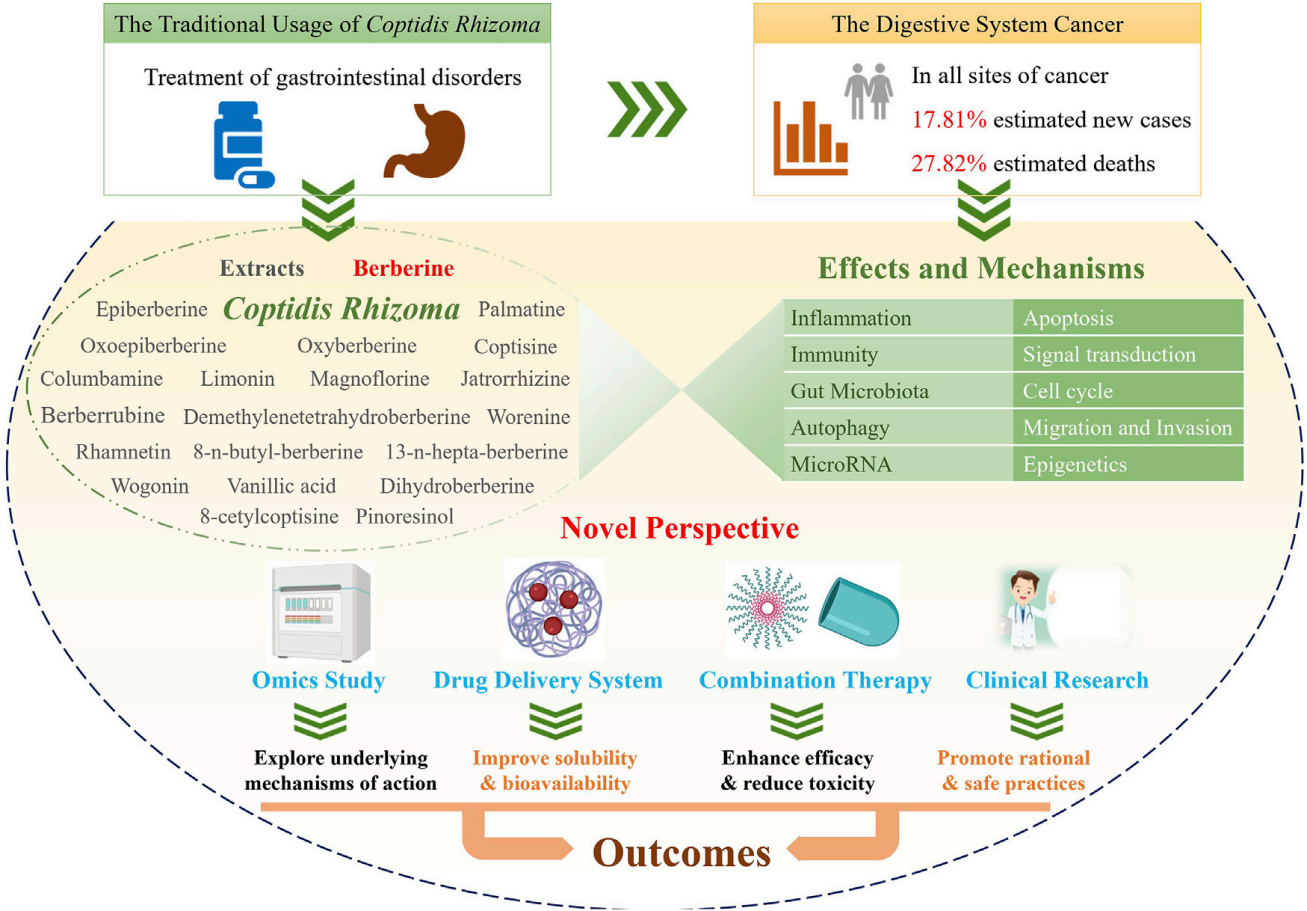
A scheme for current advance in *Coptidis Rhizoma* for gastrointestinal and other cancers. A brief scheme showing main features of *Coptidis Rhizoma* and key roles and relationship with cancers.

Taken together, Figure 2 summarizes the broad prospects of *C. Rhizoma* as an adjuvant candidate against cancers in the present review, and illustrates the rational for pre-clinical studies and clinical trials for this promising preparation.

## Glossary

5-FU: 5-fluorouracil
AgNPs: synthesized silver nanoparticles
Akt: protein kinase B
ALL: acute lymphoblastic leukemia
AMPK: adenosine monophosphate-activated protein kinase
AOM: azoxymethane
ATF4: transcription factor 4
AURKA: aurora kinase A
BBR: berberine
Bcl-2: B-cell lymphoma 2
BCRP: breast cancer resistance protein
BioReF: biological response fingerprinting
CD: Crohn’s disease
CDKs: cyclin-dependent kinases
COX-2: cyclooxygenase-2
CRC: colorectal cancer
CYP: cytochrome P450s
DEGs: differentially expressed genes
DMs: differential metabolites
DMTHB: demethylenetetrahydroberberine
DOX: doxorubicin
DSS: dextran sulfate sodium
ECM: extracellular matrix
EGCs: enteric glial cells
EGFR: epidermal growth factor receptor
EMT: epithelial-mesenchymal transition
Eph: erythropoietin-producing hepatoma
EphB4: erythropoietin-producing hepatocyte B4
ER: estrogen receptor
ERK: extracellular signal-regulated kinase
ESCC: esophageal squamous cell carcinoma
FACT: facilitates chromatin trancription
FISH: fluorescence *in situ* hybridization
G-MDSC: granulocytic-myeloid-derived suppressor cell
HCC: hepatocellular Carcinoma
HCV: hepatitis C virus
HDAC3: histone acetyltransferase 3
HDCA: histone deacetylase
HST: Hangeshashinto
HUVECs: human umbilical vein endothelial cells
IBD: inflammatory bowel disease
ICAM-1: intercellular adhesion molecular-1
IFN: interferon
IGF2: insulin-like growth factor 2
IGF2BP3: Insulin-like growth factor 2 mRNA-binding protein 3
IL: interleukin
IPA: Ingenuity Pathway Analysis
JNK: c-Jun N-terminal kinase
LC3: light chain 3
MDM2: murine double minute 2: murine double minute 2
LPS: lipopolysaccharide
LSD1: lysine-specific demethylase 1
MAPK: mitogen-activated protein kinase
MDM2: murine double minute 2: murine double minute 2
MDR: multiple drug resistance
MRP1: multidrug resistance protein 1
miR: microRNA
MMP: matrix metalloproteinase
MNEHDS: micro- and nano-encapsulated hybrid delivery system
MS: mass spectrometry
MTDH: metadherin
NAFLD: nonalcoholic fatty liver disease
NAG-1: nonsteroidal anti-inflammatory drug-activated gene-1
NEK: NIMA-related kinase
NF-κB: nuclear factor kappa-B
NLRP3: nucleotide-binding domain-like receptor protein 3
NOD: nucleotide-binding domain
NQO1: NAD(P)H quinone oxidoreductase 1
NSAID: nonsteroidal anti-inflammatory drug
NSCLC: non-small cell lung cancer
OBB: oxyberberine
OM: oral mucositis
OSM: oncostatin M
PCNA: proliferating cell nuclear antigen
PD-1: programmed cell death-1
PDCD4: programmed cell death 4
PD-L1: programmed cell death ligand-1
PDT: photodynamic therapy
PEG-lip-DOX: Pegylated liposomal doxorubicin
PGE2: prostaglandin E2
P-gp: P-glycoprotein
PI3K: phosphatidylinositol 3-kinase
PICRSt: Phylogenetic Investigation of Communities by Reconstruction of Unobserved States
PLA2G4A: Phospholipase A2a
RBPs: RNA-binding proteins
RIAISs: radiation-induced acute intestinal symptoms
ROS: reactive oxygen species
RTKs: receptor tyrosine kinases
SCFA: short-chain fatty acids
SPT16: suppressor of Ty homolog-16
SSRP1: structure-specific recognition protein 1
TGF-β1: transforming growth factor-beta-1
Th: T helper
TIMP-1: tissue inhibitor of metalloproteinase 1
TKI: tyrosine kinase inhibitor
TNF: tumor necrosis factor
TNIK: Traf2 and Nck interacting serine protein kinase
TRAIL: tumor necrosis factor related apoptosis inducing ligand
Treg cell: T regulatory cell
TS: thymidylate synthase
UC: ulcerative colitis
uPA: Urokinase plasminogen activator
VEGF: vascular endothelial growth factor
VEGFR2: vascular endothelial growth factor receptor 2
WMS: whole metagenome sequencing
XIAP: X-linked inhibitor of apoptosis protein
