# Erratum: Current Advances in Coptidis Rhizoma for Gastrointestinal and Other Cancers

**DOI:** 10.3389/fphar.2022.931882

**Published:** 2022-05-26

**Authors:** Frontiers Production Office

**Affiliations:** Frontiers Media SA, Lausanne, Switzerland

**Keywords:** coptidis rhizoma, medicinal plant, gastrointestinal cancer, omics, clinical research

Due to a production error, “Zhangfeng Zhong” was not included as an author in the published article.

The publisher apologizes for this mistake. The original version of this article has been updated.

